# Chronic mTOR inhibition in mice with rapamycin alters T, B, myeloid, and innate lymphoid cells and gut flora and prolongs life of immune‐deficient mice

**DOI:** 10.1111/acel.12380

**Published:** 2015-08-28

**Authors:** Vincent Hurez, Vinh Dao, Aijie Liu, Srilakshmi Pandeswara, Jonathan Gelfond, Lishi Sun, Molly Bergman, Carlos J. Orihuela, Veronica Galvan, Álvaro Padrón, Justin Drerup, Yang Liu, Paul Hasty, Zelton Dave Sharp, Tyler J. Curiel

**Affiliations:** ^1^ Department of MedicineUniversity of Texas Health Science CenterSan AntonioTXUSA; ^2^ Graduate School of Biomedical SciencesUniversity of Texas Health Science CenterSan AntonioTXUSA; ^3^ Department of Epidemiology and BiostatisticsUniversity of Texas Health Science CenterSan AntonioTXUSA; ^4^ Barshop Institute for Longevity and Aging StudiesUniversity of Texas Health Science CenterSan AntonioTXUSA; ^5^ Department of PhysiologyUniversity of Texas Health Science CenterSan AntonioTXUSA; ^6^ Department of Molecular MedicineInstitute of BiotechnologyUniversity of Texas Health Science CenterSan AntonioTXUSA; ^7^ Cancer Therapy & Research CenterUniversity of Texas Health Science CenterSan AntonioTXUSA

**Keywords:** immune cell differentiation, immunology, longevity, mammalian (mechanistic) target of rapamycin, metagenomics, microarray, rapamycin, transcriptomics

## Abstract

The mammalian (mechanistic) target of rapamycin (mTOR) regulates critical immune processes that remain incompletely defined. Interest in mTOR inhibitor drugs is heightened by recent demonstrations that the mTOR inhibitor rapamycin extends lifespan and healthspan in mice. Rapamycin or related analogues (rapalogues) also mitigate age‐related debilities including increasing antigen‐specific immunity, improving vaccine responses in elderly humans, and treating cancers and autoimmunity, suggesting important new clinical applications. Nonetheless, immune toxicity concerns for long‐term mTOR inhibition, particularly immunosuppression, persist. Although mTOR is pivotal to fundamental, important immune pathways, little is reported on immune effects of mTOR inhibition in lifespan or healthspan extension, or with chronic mTOR inhibitor use. We comprehensively analyzed immune effects of rapamycin as used in lifespan extension studies. Gene expression profiling found many and novel changes in genes affecting differentiation, function, homeostasis, exhaustion, cell death, and inflammation in distinct T‐ and B‐lymphocyte and myeloid cell subpopulations. Immune functions relevant to aging and inflammation, and to cancer and infections, and innate lymphoid cell effects were validated *in vitro* and *in vivo*. Rapamycin markedly prolonged lifespan and healthspan in cancer‐ and infection‐prone mice supporting disease mitigation as a mechanism for mTOR suppression‐mediated longevity extension. It modestly altered gut metagenomes, and some metagenomic effects were linked to immune outcomes. Our data show novel mTOR inhibitor immune effects meriting further studies in relation to longevity and healthspan extension.

## Introduction

Mammalian (mechanistic) target of rapamycin (mTOR) is an important cellular growth and metabolism regulator. Its signals are complex, integrating many environmental cues (e.g., growth factors, energy/nutrients) to regulate important cellular processes (e.g., autophagy, macromolecule biosynthesis) (Laplante & Sabatini, 2012). mTOR inhibitors are approved to treat some cancers, with presumed effects on cancer cell proliferation or metabolism, and with many new mTOR inhibitors in development or in clinical trials (Wander *et al*., 2011).

However, mTOR also mediates profound functional and differentiation effects on major immune cell populations, including CD8^+^ T cells (Araki *et al*., 2009), CD4^+^ regulatory and nonregulatory T cells (Chi, 2012), and myeloid and B cells (Weichhart & Saemann, 2009). Immunosuppression is a major concern with long‐term mTOR inhibition, but recent data show that rapamycin can boost antipathogen (Keating *et al*., 2013) and antitumor immunity (Diken *et al*., 2013) and was not detrimentally immunosuppressive in a mouse organ transplant model (Ferrer *et al*., 2010). Further, a recent report (Mannick *et al*., 2014) shows that mTOR inhibition with the rapalogue, everolimus, improves influenza vaccine responses in elderly humans.

Caloric restriction also inhibits mTOR, and extends lifespan in many organisms including yeast, flies, worms, mice, and primates (Colman *et al*., 2014). As caloric restriction is unlikely to be popular or practical in large human populations and as human genetic manipulations for lifespan or healthspan extension are unlikely soon, pharmacologic mTOR inhibition has received wide attention. The best studied pharmacologic mTOR inhibitor in lifespan and healthspan extension studies is microencapsulated rapamycin (eRapa) formulated to improve its pharmacokinetics (Harrison *et al*., 2009). mTOR forms 2 complexes, mTORC1 and mTORC2. Rapamycin is considered primarily an mTORC1 inhibitor (Laplante & Sabatini, 2012) but can also inhibit mTORC2. Recent studies distinguish mTORC1 versus mTORC2 effects on lifespan (Selman *et al*., 2009) in addition to effects on cell proliferation (Dowling *et al*., 2010), metabolism (Sengupta *et al*., 2010; Lamming *et al*., 2012), protein translation (Thoreen *et al*., 2012), immunity (Chi, 2012), and other processes (Yu *et al*., 2010; Shimobayashi & Hall, 2014).

Surprisingly, only few limited reports of immune effects of mTOR inhibitors in longevity or healthspan studies have been published (Neff *et al*., 2013), prompting our studies. We describe long‐term effects of eRapa in young and aged C57BL/6 mice on gene expression, function, and differentiation of T and B lymphocytes, myeloid cells, and innate lymphoid cells (ILCs), and effects on gut metagenomes. Our findings identify novel aspects of, and afford new insights into mTOR effects on important immune cells and show immune effects of mTOR inhibition that could contribute to longevity and healthspan extension and help explain anticancer effects and mitigation of other age‐related debilities. Other immune effects suggest that further studies of mTOR influences on immunity in distinct aspects of age‐related diseases and other scenarios are warranted.

## Results

### eRapa alters gene expression in many immune cell populations and preferentially reduces memory T lymphocytes

**Figure 1:**
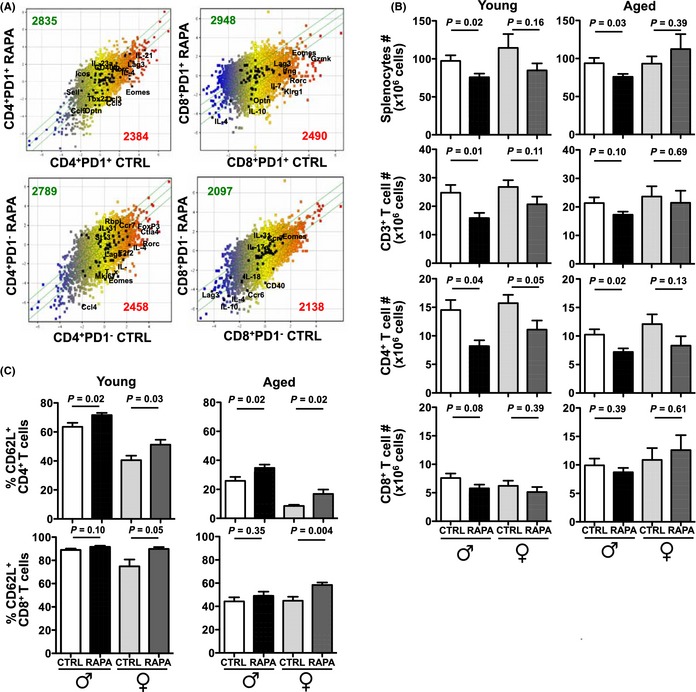
eRapa alters gene expression in immune cells resulting in preferential reduction in memory T lymphocytes. (A) Scatterplots of normalized gene expression values (Log2 scale) in sorted CD4^+^ PD‐1^+^, CD4^+^ PD‐1^−^, CD8^+^ PD‐1^+^, and CD8^+^ PD‐1^−^ T cells comparing cells from eRapa‐fed 22‐month‐old male BL6 mice (RAPA,* Y*‐axis) versus Eudragit control (CTRL,* X*‐axis) mice (mean, *n* = 3 mice/group). The numbers of genes upregulated (green) or downregulated (red) by more than twofold in eRapa versus CTRL are indicated. Representative genes are annotated. (B) Numbers of total spleen cells, CD3^+^, CD4^+^, and CD8^+^ T cells from young (8 months old) and aged (24–25 months old) male and female C57BL/6 mice on Eudragit (CTRL) or eRapa (RAPA) chow for 6 months (*n* = 10‐15 mice/group). (C) Frequency of CD62L^+^ cells in CD4^+^ and CD8^+^ splenic T cells from young (8 months old) and aged (24–25 months old) male and female C57BL/6 mice on Eudragit (CTRL) or eRapa (RAPA) chow for 6 months (*n* = 10–15 mice/group). All error bars represent standard error of mean (SEM).

Although eRapa effects in lifespan extension are greater in females (Harrison *et al*., 2009), we did not see obvious trends in sexual immune dimorphisms in our immune studies although some parameters were significantly altered only in males. We thus isolated spleen cells from 22‐month‐old male mice fed eRapa (14 ppm, the originally reported life‐extending dose) (Harrison *et al*., 2009) or control for 12 months (three mice per group) and used multiparameter flow cytometry to sort seven important immune cell populations to high purity: CD4^+^PD‐1^+^, CD4^+^PD‐1^−^, CD8^+^PD‐1^+^, and CD8^+^PD‐1^−^ T cells; B220^+^ B cells; CD11b^+^CD11c^−^ myeloid cells; and CD11b^+^CD11c^+^ dendritic cells to assess gene expression (gating strategies: Figs S1–S5). Overall, eRapa upregulated or downregulated the average expression of ~2000–3000 genes >twofold in these populations (Fig. 1A and data not shown). Thus, long‐term eRapa as used to extend lifespan or healthspan exerts profound effects on many important lymphoid and myeloid immune cell populations.

eRapa decreased numbers of total spleen immune cells, consistent with known rapamycin cytopenic effects (Sofroniadou & Goldsmith, 2011). CD3^+^ T cells were decreased, preferentially affecting CD4^+^ T cells in young and aged mice of both sexes on eRapa for 6 months (Fig. 1B), correlating with changes in genes affecting T‐cell proliferation and homeostasis (e.g., *il2*,* il7R*; Table S1). eRapa reduced T‐cell memory/activation markers (e.g., *cd44*) and increased T cell expressing naïve phenotypes (e.g., increased *cd62 l*,* ccr7*; Table S1).

Flow cytometry confirmed eRapa increased CD62L^+^CD4^+^ and CD62L^+^CD8^+^ spleen T‐cell prevalence to a greater extent in aged mice with lower basal CD62L^+^ T cells (Fig. 1C), reflecting age‐related CD62L^−^ memory T‐cell accumulation (Sun *et al*., 2012). Similar CD4^+^ T‐cell reductions and increased CD62L^+^ T cells were noted in Peyer's patches (Fig. S6A). Thus, eRapa appears to mitigate age‐related decline in naïve T cells.

### Long‐term eRapa alters T‐cell trafficking

Increased CD62L and CCR7 (Table S1) suggested long‐term eRapa could affect T‐cell trafficking. We thus injected T cells from eRapa‐ or Eudragit‐treated mice into untreated young recipients and analyzed proportions of transferred cells in various organs. T cells from eRapa‐treated mice, particularly CD8^+^ T cells, migrated preferentially to lymph nodes and Peyer's patches, consistent with their higher CD62L and CCR7 expression compared to Eudragit‐fed mice, while no differential distribution was seen in blood (Fig. S6B), consistent with *in vitro* studies of rapamycin‐treated CD8^+^ T cells (Sinclair *et al*., 2008).

### Long‐term eRapa reduces PD‐1^+^ and exhausted T cells

**Figure 2:**
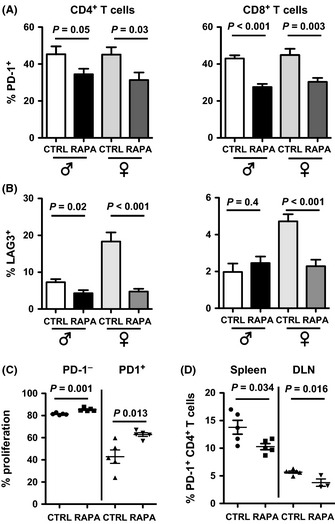
eRapa reduces T‐cell exhaustion markers. (A) PD‐1^+^ cell prevalence in CD4^+^ and CD8^+^ splenic T cells from 24‐ to 25‐month‐old male and female C57BL/6 mice on Eudragit (CTRL) or eRapa (RAPA) chow for 6 months (*n* = 10–15 mice/group). (B) LAG3^+^ cell prevalence in CD4^+^ and CD8^+^ splenic T cells from 22‐ to 24‐month‐old male and female C57BL/6 mice on Eudragit or eRapa chow for 12 months (*n* = 7–11 mice/group). (C) Spleen CD4^+^ T cells from naïve BL6 mice were stimulated *in vitro* with anti‐CD3/anti‐CD28 antibodies for 2 days plus 5 ng/mL rapamycin (RAPA) or DMSO control (CTRL). MFI, mean fluorescence intensity. (D) eRapa‐fed, young mice challenged with subcutaneous B16F10 melanoma cells have reduced PD‐1^+^ CD4^+^ T cells in spleen and tumor‐draining lymph nodes (DLN). *n* = 5 mice/group.

T cells become exhausted with age, losing functionality for unclear reasons that could include long‐term antigenic stimulation or chronic inflammation (Lages *et al*., 2010). PD‐1 is an exhausted T‐cell marker (Barber *et al*., 2006). Long‐term eRapa significantly reduced PD‐1^+^CD4^+^ and PD‐1^+^CD8^+^ T‐cell prevalence in aged mice (Fig. 2A), suggesting reduced exhaustion. Young mice had <3% PD‐1^+^ T cells at baseline that were unaltered by eRapa (not shown). In PD‐1^+^ and PD‐1^−^ aged T cells, long‐term eRapa reduced expression of *pd1* and other genes associated with T‐cell exhaustion (e.g., *klrg1, lag3, havcr2* (Tim‐3); Table S1). Reduced LAG3 in aged T cells was confirmed by flow cytometry (Fig. 2B). The *mki67* (Ki‐67) proliferation marker was strongly reduced by eRapa in all T‐cell subsets. The *cd69* acute activation marker was slightly reduced in PD1^−^ T cells and unchanged in PD‐1^+^ T cells (Table S1). To test for the effect of rapamycin on functional T‐cell exhaustion, we sorted PD‐1^+^ and PD‐1^−^ T cells from mice fed eRapa or control diet for 6 months (five mice per group), stimulated them for 4 days with anti‐CD3/CD28 antibodies, and assessed proliferation. PD‐1^−^ T cells from eRapa‐ and control‐fed mice proliferated equally well, whereas the PD‐1^+^ T cells from control mice proliferated poorly, consistent with exhaustion. This proliferative defect was partially reversed in PD‐1^+^ T cells from eRapa mice (Fig. 2C) consistent with reversal of exhaustion.

Cancers induce T‐cell exhaustion even in young hosts (Wherry, 2011). We placed 8‐week‐old mice on eRapa or control for 3 months, challenged with subcutaneous B16F10 melanoma, and showed that eRapa significantly reduced T‐cell PD‐1 expression (Fig. 2D) without affecting tumor growth, regulatory T cells, or myeloid‐derived suppressor cells (Hasty *et al*., 2014). Thus, oral rapamycin reduces T‐cell PD‐1, suggesting reduced exhaustion with aging and in cancer.

### Long‐term eRapa promotes immune cell lipogenic genes and inhibits glycolytic genes

There was a trend for increased lipogenic metabolic genes and decreased glycolytic genes in CD4^+^ and CD8^+^ T cells that regulate their differentiation and function (Table S1) consistent with known rapamycin effects (Shimobayashi & Hall, 2014).

### Long‐term eRapa modulates CD4^+^ and CD8^+^ T‐cell differentiation

**Figure 3:**
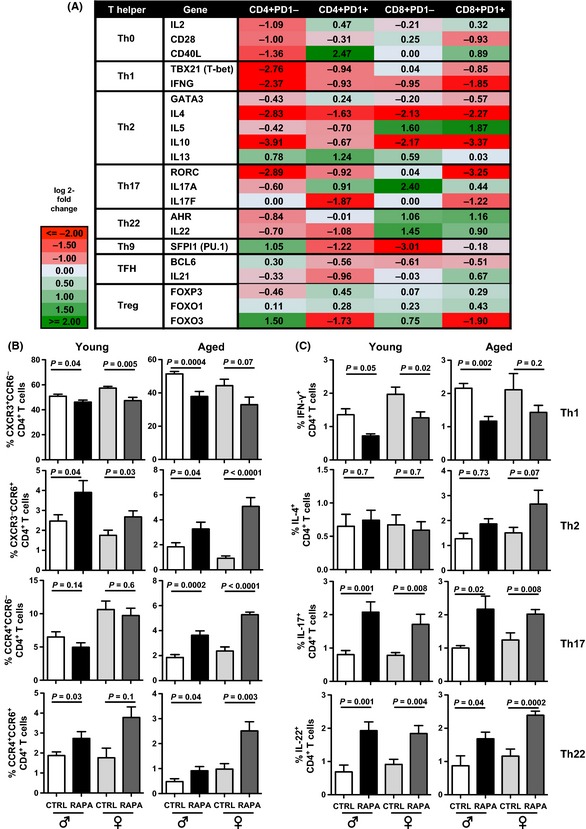
eRapa alters T helper (Th) pathway differentiation. (A) Log2 fold‐changes (ratio of eRapa over CTRL normalized gene expression) in genes characteristic of Th/cytotoxic subsets in CD4^+^ and CD8^+^ T‐cell subpopulations. Analyses of spleen cells sorted from 22‐month‐old male BL6 mice (*n* = 3/group). (B) Chemokine receptor‐expressing cell prevalence in CD4^+^ splenic T cells from young (8 months) and aged (24–25 months) male and female C57BL/6 mice on Eudragit (CTRL) or eRapa (RAPA) chow for 6 months (*n* = 10–15 mice/group). (C) Cytokine‐expressing cell prevalence in CD4^+^ splenic T cells. Cytokines detected by intracellular flow cytometry after 18‐h stimulation with anti‐CD3/CD28 beads. All error bars represent SEM.

mTOR is critical in Th1, Th2, and Th17 differentiation (Delgoffe *et al*., 2011; Powell *et al*., 2012) and has unreported effects on other Th differentiation pathways. Global gene pathway analysis of CD4^+^PD‐1^−^ T cells confirmed significant eRapa‐mediated modulation of Th differentiation (Fig. S7). In CD4^+^PD‐1^−^ T cells, eRapa reduced *tbx21* (Th1), *gata3* (Th2), *rorc* (Th17), and *ahr* (Th22) and increased *pu.1* (Th9) and *bcl6* (T_FH_) (Fig. 3A). Expressions of these transcription factors and associated cytokines were also modulated in all other T‐cell populations analyzed. eRapa increased Th17 and Th22 signature genes in CD8^+^PD‐1^−^ cells (Fig. 3A) and affected chemokine receptor genes distinguishing Th subsets (e.g., decreased CXCR3 and CCR5 in all T‐cell populations, and increased CCR6, CCR4, and CCR10 in CD4^+^PD‐1^+^ T cells; Table S2).

We corroborated genomic data by flow cytometry and found that long‐term eRapa reduced spleen CD4^+^ T cells with a Th1 signature (CD4^+^CXCR3^+^) and increased Th17/Th22 signatures (CD4^+^CCR4^+^CCR6^+^) in spleen and Peyer's patches (Figs 3B and S8A). Th2 (CD4^+^CCR4^+^CCR6^−^) signatures increased only in aged mice, the only differential age effect on Th polarization detected, corroborated by increased IL‐4 expression (Fig. 3C). Similar trends were observed in CD8^+^ T cells (Fig. S8B). Flow cytometry confirmed functional Th1, Th17, and Th22 skewing as reduced IFN‐γ^+^CD4^+^ (and IFN‐γ^+^CD8^+^) T cells and increased IL‐17^+^ and IL‐22^+^ CD4^+^ T cells in spleen and Peyer's patches (Figs 3C and S8C). Similar trends for all effects were observed in CD8^+^ T cells (Fig. S8D). Differential cytokine gene expression in eRapa‐treated mice was observed with consistent decreases in *ifng*,* il4*, and *il10*, while other Th cytokine genes (e.g., *il17, il22*) were variably affected by eRapa in distinct T‐cell subsets (Fig. 3A).

### Long‐term eRapa alters regulatory T‐cell (Treg) prevalence but not function

**Figure 4:**
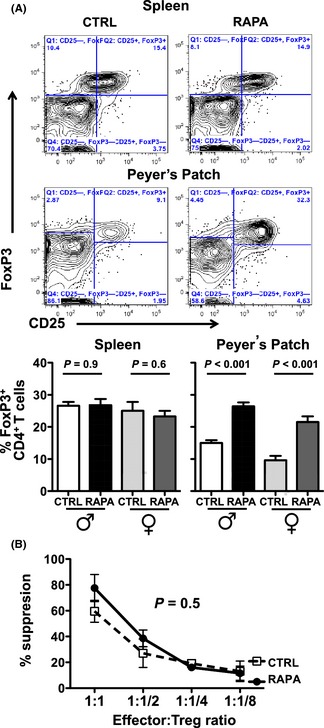
eRapa effect on Treg numbers and function. Naïve male and female C57BL/6 mice were given eRapa (RAPA) or control (CTRL) chow for 21 months starting at 4 months of age. (A) Representative dot plots and frequency of Foxp3^+^ among total CD4^+^ T cells in spleen, mesenteric lymph nodes (MLN) and Peyer's patches (*n* = 6 mice/group). All error bars represent SEM. (B) Spleen Treg suppression, assessed in triplicate at various CD4^+^ effector:Treg ratios. *P*‐value, two‐way anova.

Rapamycin promotes regulatory Treg generation *in vitro* in mouse and human cell cultures (Golovina *et al*., 2011), with incompletely understood *in vivo* Treg effects. Long‐term eRapa does not increase mouse blood Tregs (Goldberg *et al*., 2015). Treg prevalence in spleen and mesenteric lymph nodes of mice started on eRapa at age 6 months for 19 months was unchanged, but the prevalence increased in Peyer's patches (Fig. 4A) potentially from higher rapamycin concentrations in intestines versus other tissues as we reported (Hasty *et al*., 2014). Nonetheless, suppressive function of CD4^+^CD25^hi^ Tregs from spleens of 10‐month‐old mice fed eRapa for 8 months was not significantly altered (Fig. 4B). Insufficient cells were obtained from Peyer's patches for functional assessments.

### eRapa remarkably prolongs lifespan in the absence of lymphocytes or IFN‐γ

**Figure 5:**
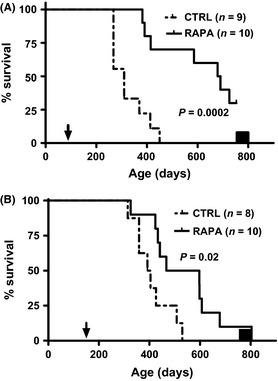
eRapa increases lifespan of immunocompromised cancer‐prone mice. *(*A) RAG2 KO (knockout) mice given eRapa (RAPA) or Eudragit control (CTRL) diet starting at 3 months of age (arrowhead). *P*‐value from log‐rank test. RAG2, recombinase‐activating gene 2. Median survival, Eudragit, 310 days; and eRapa, 685 days. (B) IFN‐γ KO mice given eRapa or Eudragit control diet starting at 5 months of age (arrowhead). *P*‐value from log‐rank test. Median survival, Eudragit, 398 days and eRapa, 532 days. Black box indicates median lifespan range (750‐800 days) of C57BL/6J mice.

We showed that reduced neoplasia is an eRapa‐mediated longevity extension mechanism in spontaneous neoplasia models (Livi *et al*., 2013; Hasty *et al*., 2014) and potentially in wild‐type mice (Miller *et al*., 2011). T cells and IFN‐γ mediate cancer immune surveillance (Mittal *et al*., 2014). eRapa mediated remarkably potent lifespan (Fig. 5) and healthspan (Videos S1 and S2) extension in RAG2^−/−^ mice lacking T and B lymphocytes, with survival approaching that of untreated wild‐type mice. It also significantly increased lifespan in IFN‐γ^−/−^ mice (Fig. 5), each exceeding the typical ~10% median lifespan extension of eRapa and other known pharmacologic lifespan‐extending agents in wild‐type mice (Zhang *et al*., 2014) by more than 3.3‐ or 12‐fold for IFN‐γ^−/−^ or RAG2^−/−^ mice, respectively.

### Long‐term eRapa increases myeloid cell population prevalence

Long‐term eRapa significantly increased the prevalence but not the absolute number of CD11b^+^CD11c^−^ monocytes/macrophages, conventional CD11b^+^CD11c^+^ dendritic cells, and cells with a CD11b^+^Gr1^hi^ myeloid‐derived suppressor cell phenotype in young and aged mouse spleens (Fig. S9A). We assessed myeloid cell genes affecting T‐cell maturation and differentiation and found that eRapa increased *ifna, il1b, tgfb1, cd80*, and *cd86* while decreasing *ifng, il4, il6*, and *il10* (Table S3). We differentiated myeloid cells *in vitro* in the presence of rapamycin at 5 ng/mL (similar to *in vivo* mouse levels). In contrast to *in vivo* effects, rapamycin significantly reduced CD11b^+^CD11c^+^ dendritic cell prevalence although they acquired activated phenotypes (Fig. S10A) similar to *in vivo* and genomic data.

### Rapamycin‐conditioned dendritic cells alter Th polarization

*In vitro* studies suggest that rapamycin reduces Th17 polarization through direct T‐cell effects (Kopf *et al*., 2007) contrasting with our *in vivo* data (Fig. 3). Indirect Th1 and Th17 polarization effects through CD11b^+^ myeloid cells are described *in vivo* (Iwasaki & Medzhitov, 2010). After maturation with either lipopolysaccharide or IL‐1β/TNF‐α, rapamycin‐conditioned dendritic cells recapitulated *in vivo* eRapa effects by reducing T‐cell Th1 polarization and augmenting Th17 polarization (Fig. S10B). Genomic data assessing myeloid cell factors support this effect (e.g., increased *il6, il21, il23*; Table S3). These data accord with coordinated eRapa effects on distinct cells *in vivo* to skew Th polarization directly and indirectly.

### Long‐term eRapa differentially affects B‐cell subpopulation prevalence and increases naïve phenotype

Long‐term eRapa increased B220^+^ B‐cell prevalence in spleen and bone marrow of young and aged mice (Fig. S9B) without significant change in absolute numbers in spleen. In spleen, it specifically increased IgM^+^AA4.1^−^CD23^+/−^ follicular and marginal zone B cells and decreased IgM^+^AA4.1^+^CD23^**−**^ transitional 1 (T1) B‐cell prevalence (Fig. S9C). Genes regulating B‐cell homeostasis or differentiation were significantly affected. For example, *cd27, il7r, cd93* (AA4.1), and *cd43* were strongly downregulated by eRapa, whereas *cd19, cd20, cd21*, and *cd23* were upregulated (Table S4), consistent with flow cytometry data and suggesting eRapa‐mediated preservation of naïve B‐cell phenotypes.

### Long‐term eRapa alters distinct cell death pathways, related inflammation, and autophagy

Age is associated with increased generalized inflammation of obscure origin thought to contribute to age‐related debilities and premature death (Franceschi & Campisi, 2014). Rapamycin can alter apoptosis and necrosis that influence inflammation, with differential effects by tissue and model (Fielhaber *et al*., 2012). eRapa‐fed mice exhibited reduced apoptosis‐ (e.g., *bak, casp3, fas, fasl*) and necroptosis‐related (e.g., *ripk1, tradd*) genes in all immune cells tested (Table S5). eRapa modulated inflammasome‐related genes variably in most immune cells studied (Table S5). We found a dose‐dependent decrease in anti‐inflammatory A20, but an increase in IκB‐α in lungs of 22‐month‐old eRapa‐treated UM‐Het3 mice on eRapa for 13 months suggesting complex effects of rapamycin on both pro‐ and anti‐inflammatory pathways (Fig. S10C). We could not assess mTOR signaling by gene arrays, as key mediators are phosphoproteins, but demonstrated cell‐specific effects on genes downstream of mTOR signaling (e.g., protein translation genes) and on autophagy‐related genes in eRapa‐treated mice (Table S6).

### Long‐term eRapa increases innate lymphoid cell populations and functions

**Figure 6:**
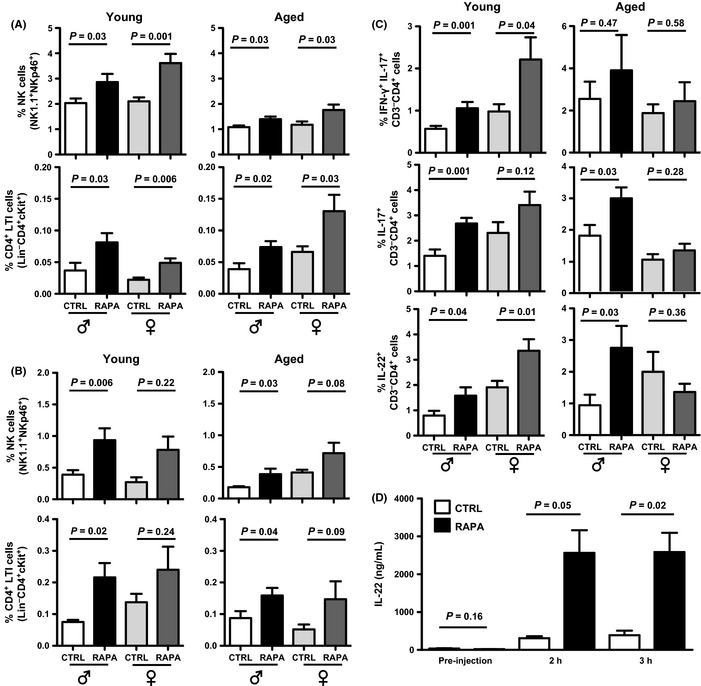
eRapa increases innate lymphoid cells and flagellin‐induced IL‐22. (A *–*B) NK1.1^+^ NKp46^+^ cell and Lin^−^ cKIT ^+^ CD4^+^ cell prevalence in total spleen (A) and Peyer's patches (B) from young (8 months old) and aged (24‐25 months old) male and female C57BL/6 mice on Eudragit (CTRL) or eRapa (RAPA) chow for 6 months (*n* = 8‐15 mice/group). Lin, lineage (CD3, B220, CD11b, CD11c, NK1.1). (C) eRapa increases spleen group 3 ILC IL‐17 and IL‐22. Cytokine‐expressing cell prevalence in CD3^−^ CD4^+^ LTI‐like cells from spleens. Cytokines detected by intracellular flow cytometry after 18‐h stimulation with anti‐CD3/CD28 beads. (D) Wild‐type BL6 mice on Eudragit (CTRL) or eRapa (RAPA) were injected with 1 μg flagellin. Serum assayed for IL‐22 at indicated time points (*N* = 3‐7/group). *P*‐values, unpaired *t*‐test. LTI, lymphoid tissue inducer. All error bars represent S.E.M.

Three ILC groups are defined based on functional attributes and nuclear transcription factor expression (Spits *et al*., 2013). Their *in vivo* regulation is incompletely understood. Our ILC studies were carried out before these unifying ILC groups were described. Chronic eRapa increased NK1.1^+^NKp46^+^ group 1 ILCs (natural killer cells) and lineage^−^CD4^+^c‐kit^+^ group 3 ILCs (also called lymphoid tissue inducers) in spleen and Peyer's patches (Fig. 6A,B). We confirmed group 3 ILC function in spleen and Peyer's patches by IL‐17 and IL‐22 production from these cells (Figs 6C and S11A).

### eRapa promotes flagellin‐induced IL‐22 without *Citrobacter rodentium* protection

We challenged 8‐month‐old mice on eRapa for 6 months with flagel‐lin, which augments IL‐23‐driven IL‐22 from intestinal group 3 ILC (Basu *et al*., 2012). Mice on eRapa experienced eightfold increased flagellin‐driven serum IL‐22 versus Eudragit (Fig. 6D). As IL‐22‐producing group 3 ILCs protect from intestinal bacterial infection, we challenged RAG2^−/−^ mice on eRapa or Eudragit for 1 month with the flagellated bacterium *C. rodentium* as described (Basu *et al*., 2012). eRapa did not protect from chronic infection as determined by survival, weight change, and colon length (Fig. S11B‐D).

### Long‐term eRapa has minor gut metagenome effects

Immune cells and commensal gut flora regulate each other reciprocally (Sonnenberg & Artis, 2012). Further, rapamycin has antimicrobial effects. We therefore tested whether long‐term eRapa altered gut flora. Analysis of fecal metagenomes in 19‐month‐old mice fed eRapa or Eudragit for 12 months identified minor global population changes without sex differences. Four operational taxonomic units were significantly modulated in eRapa mice, belonging to Firmicutes, Acidobacteria**,** and Bacteroidetes (Fig. S11E). Changes in whole microbiome significantly correlated with IL‐17^+^CD4^+^ Peyer's patch T cells (*P* = 0.026) and CD62L^+^CD4^+^ and CD62L^+^CD8^+^ splenic T cells (*P* = 0.001 and 0.037, respectively, Adonis tests).

## Discussion

mTOR mediates significant immune effects that remain incompletely elucidated. As mTOR inhibitors have entered clinical practice as anticancer drugs and are under evaluation as vaccine adjuvants, adjuncts to treating autoimmune disorders and to extend lifespan and healthspan, understanding the range of mTOR effects on immunity is crucial. Recent evidence shows that rapamycin specifically, and mTOR inhibit‐ion generally, improves important immune functions (Weichhart & Saemann, 2009; Ferrer *et al*., 2010; Chi, 2012; Diken *et al*., 2013; Keating *et al*., 2013; Zeng *et al*., 2013). We show that eRapa profoundly affected gene expression in seven important immune populations and suggest how these immune contributions could contribute to anticancer, lifespan extension, or healthspan extension, among other considerations.

As the population ages, age‐related debilities have increased, contributing significantly to societal, health, and financial burdens. There is a considerable interest in strategies that increase lifespan, which ideally will also extend healthspan. Rapamycin is the first pharmacologic agent that improves mammalian lifespan. It prolongs lifespan and healthspan even when started late in life and in mixed genetic backgrounds and improves healthspan (Harrison *et al*., 2009; Hasty *et al*., 2014). Thus, rapamycin is a leading pharmacologic candidate to extend human lifespan and healthspan. However, it was developed as an immunosuppressive agent (Hasty *et al*., 2014), raising legitimate safety concerns (Lamming *et al*., 2013). Our extensive data show that long‐term rapamycin is a potent immune modulator and not clinically immunosuppressive in numerous scenarios.

Notably, long‐term eRapa reduced phenotypically exhausted T cells that can be functionally impaired and contribute to age‐related immune dysfunction (Lages *et al*., 2010). Gene array data showed that PD‐1^+^ cells in eRapa‐treated mice had reduced signature exhaustion gene expression suggesting better function versus PD‐1^+^ control T cells. We predict these cells contribute to improved eRapa‐mediated cancer and pathogen protection, and lifespan and healthspan extension. Although mTOR suppression could promote Treg generation, eRapa did not increase Treg numbers in various organs consistent with previous studies (Neff *et al*., 2013) but did increase their prevalence in Peyer's patches. We further found no evidence supporting increased Treg suppressive function, although altered function in Peyer's patches remains untested.

A recent human study demonstrated that the rapalogue everolimus improved antigen‐specific humoral immunity to influenza vaccine in elderly humans (Mannick *et al*., 2014). As much work also supports rapamycin‐mediated improvements in T‐cell functions (Ferrer *et al*., 2010), including improved antigen‐specific T‐cell memory (Araki *et al*., 2009), much additional investigation is merited. Our demonstrations of preserved naïve phenotypes in B and T cells in aged mice on eRapa support potential benefits for antigen‐specific immunity and could be related to its reported rejuvenating effect on hematopoietic stem cells (Chen *et al*., 2009).

We previously demonstrated significant lifespan extension in neoplasia‐prone Rb^+/−^ and *Apc* ^Min/+^ mice (Livi *et al*., 2013; Hasty *et al*., 2014). We interpret the eRapa‐mediated 3.3‐ to 12‐fold augmented lifespan extension of IFN‐γ^−/−^ and RAG2^−/−^ mice versus modest lifespan extension in syngeneic BL6 mice (Zhang *et al*., 2014) as consistent with cancer and infection prevention as lifespan extension mechanisms of eRapa, seen in exaggerated form in these mice that are highly susceptible to cancers and infections. Given the importance of cancer to mortality, and the considerable immune modulating effects of eRapa, much additional work is needed. These data are also important to understand the mechanisms of the many rapalogues now in cancer clinical trials (Shi & August, 2008).

Our gene array data suggest previously unreported eRapa‐mediated effects on skewing Th9, Th22, and T_FH_ differentiation. Flow cytometry data corroborate Th22 skewing, but additional work is required to ascertain Th9 and T_FH_ effects, as functional studies did not always corroborate gene expression data. Lack of corroboration could be due to the specific cell populations we studied.

We found a previously unidentified eRapa effect on boosting basal and induced group 3 ILC IL‐22, but with poor protection from *C. rodentium* infection, possibly reflecting insufficient eRapa‐mediated IL‐22 or lack of eRapa‐induced Th22 cells, protective in long‐term *C. rodentium,* but absent in the RAG2^−/−^ mice studied. ILCs mediate innate defense against infections and promote epithelial surface health (Hazenberg & Spits, 2014). The concept that long‐term eRapa improves longevity or healthspan by mitigating infections or tissue damage deserves consideration.

Our data show eRapa‐mediated increased group 1 and 3 ILCs in distinct organs, suggesting mTOR‐mediated homeostasis control. Given our poor understanding of *in vivo* regulation of ILC homeostasis, this novel finding bears additional investigation. We predicted significant gut metagenome effects. Instead, long‐term eRapa produced modest global intestinal metagenomic changes and significant changes in few specific operational taxonomic units. Nonetheless, specific effects could be important to understand as we found an association between eRapa‐mediated gut metagenomes and immune effects, and intestinal bacterial products can extend lifespan, including through mTOR (Chin *et al*., 2014).

Myeloid cells can contribute to inflammation. eRapa did not affect myeloid cell differentiation appreciably *in vivo* but increased myeloid cell activation. Rapamycin skewed myeloid differentiation *in vitro*. These data demonstrate previously unknown myeloid cell effects of rapamycin and suggest a complex integration of effects *in vivo*. We previously reported that aged Tregs differentially controlled myeloid cell numbers in tumor models in aged mice (Hurez *et al*., 2012) and that aged Tregs have a specific defect in regulating intestinal IL‐17 production (Sun *et al*., 2012). eRapa effects on Tregs regulating myeloid cell differentiation or activation *in vivo* remain to be studied.

Cell turnover and necrosis could contribute to age‐related inflammation (Franceschi & Campisi, 2014). eRapa increased inflammatory pyroptosis genes, but pyroptosis contributions to any eRapa‐mediated inflammation are unclear. Rapamycin and mTOR have variable effects on apoptosis and necrosis depending on tissue and other factors. eRapa also decreased anti‐inflammatory A20 while increasing IκB‐α in lungs. As age‐related inflammation can contribute to debilities and anti‐inflammatory agents are proposed to mitigate them (Franceschi & Campisi, 2014), it will be important to understand the net effects of eRapa‐mediated pro‐inflammatory and anti‐inflammatory properties, to understand their tissue‐specific effects and to place these in the context of contributions to lifespan or healthspan.

eRapa significantly increased total B‐cell prevalence in aged mice, including B cells with a naïve phenotype. Such effects could improve antipathogen immunity by allowing novel immune responses later in life or augment vaccine efficacy against novel antigens such as hepatitis B. Improved anti‐influenza humoral immunity with everolimus after vaccination of elderly patients (Mannick *et al*., 2014) supports benefits for antipathogen immunity. mTOR and rapalogue effects on B cells are little studied (Xu *et al*., 2012) but merit additional attention.

eRapa‐mediated metabolic effects in immune cells accord with the effects demonstrated in other tissues, but how these relate to immune outcomes in current studies and whether these are due to mTORC1 or mTORC2 signals are unknown. Promoting autophagy, a known rapamycin effect, can augment immune memory, yet we found increased naïve T and B lymphocytes with long‐term eRapa. Given the extensive influences of mTOR on metabolism and the growing appreciation for links to immune outcomes (Xu *et al*., 2012; Zeng *et al*., 2013), mTOR metabolic effects in clinical and immune outcomes likely will now receive much deserved attention.

There are caveats and considerations regarding our data. First, we did not discriminate short‐term versus long‐term eRapa effects as we were interested in effects in longevity and healthspan extension, where long‐term administration is likely. However, an interesting but unanswered question is whether long‐term rapamycin administration is required for longevity extension. A recent report showed that once‐weekly parenteral rapamycin prolonged survival in obese mice (Leontieva *et al*., 2014). As details are better understood, immune studies on different drug schedules should be considered. There could be significant differences as long‐term rapamycin can inhibit mTORC2 in addition to mTORC1. Second, we focused on the original 14 ppm life‐extending eRapa dose (Harrison *et al*., 2009). Recent studies show that eRapa dose‐dependently extends male and female lifespan in genetically heterogeneous mice (Miller *et al*., 2014), and we showed that eRapa dose‐dependently extends lifespan and healthspan in *Apc* ^Min/+^ mice that die from intestinal tumor bleeding (Hasty *et al*., 2014). Thus, distinct doses (and schedules) could be identified for specific outcomes. Third, eRapa extends life in females greater than males (Harrison *et al*., 2009), but we identified few significant eRapa‐mediated immune sexual dimorphisms despite extensive analyses. Although we validated many immune outcomes in both sexes, sexual dimorphisms could nonetheless occur, as in rapamycin proteasome effects (Rodriguez *et al*., 2014). Fourth, eRapa effects extremely high rapamycin concentrations in intestines, with lower concentrations in other tissues and blood (Hasty *et al*., 2014). How these concentrations and distributions compare to other rapamycin formulations and the potentially distinct immune consequences is unknown. Finally, we focused on selected lymphoid tissues (spleen, lymph nodes, Peyer's patches) in naïve mice, as these are important in orchestrating immune responses. In inflammation or distinct organs, immune effects could differ. For example, we found that eRapa reduced IFN‐γ in T cells in skin inflamed with phorbol myristate acetate (Dao *et al.,* unpublished results), which could reflect a skin effect, an inflammation effect, or other factors.

There is a great and growing interest in pharmacologic approaches to longevity and healthspan extension. Among a handful of agents now confirmed to extend life or healthspan, rapamycin is a leading candidate. Given the wide range of effects of mTOR on distinct immune cells and outcomes, and the importance of inflammation in age‐related debilities, our data sets should assist a wide range of investigations into mechanisms of rapamycin and rapalogues.

## Experimental procedures

### Mice and diets

Mice were housed in microisolators in specific pathogen‐free conditions and treated according to National Institutes of Health guidelines for the use of experimental animals and in accordance with institutional policy. Four‐ to eight‐week‐old (Jackson Laboratories, Bar Harbor, ME, USA) and 17‐ to 19‐month‐old (NIA) C57BL/6 (BL6) mice were used for all studies except longevity, in which case IFN‐γ^−/−^ and RAG2^−/−^ mice from Jackson Laboratories were used starting at ages 6‐10 weeks. Mouse chow with encapsulated rapamycin was made by TestDiet (Richmond, IN, USA) and delivers ~2.24 mg rapamycin/kg/day in Purina 5LG6 chow (Harrison *et al*., 2009). The Eudragit control was identical except without rapamycin. This eRapa formulation reliably mediates serum rapamycin levels of ~5 ‐15 ng/mL, which we confirmed in the major cohorts of aged mice for these studies (not shown). This rapamycin level also suppresses mTORC1 signaling (rpS6, 4EBP‐1, and S6K) in fat, intestines, and liver as we and others described (Harrison *et al*., 2009; Hasty *et al*., 2014). In survival studies, mice were allowed to live out normal lifespan until spontaneous death, or were sacrificed for distress according to defined criteria as we reported (Harrison *et al*., 2009; Hasty *et al*., 2014).

### Flow cytometry

We isolated and stained cells and performed cell sorts as previously described (Hurez *et al*., 2012). Data were acquired on an LSRII (BD Biosciences, San Jose, CA, USA) and analyzed with FlowJo (Tree Star, Ashland, OR, USA). Fluorochrome‐conjugated antibodies were from BD Biosciences, eBioscience, Caltag Laboratories, Biolegend, or R&D.

### Whole‐genome gene expression

Spleen cells from 22‐month‐old male mice fed eRapa or Eudragit for 12 months (three mice/group) were sorted to high purity on a BD FACSAria. Total cellular RNA was purified using Quick‐RNA MicroPrep (Zymo Research, Irvine, CA, USA) for each individual immune population sorted from each individual mouse. cRNA probe preparation and hybridization to Illumina MouseWG‐6 version 2 BeadChips were carried out with standard Illumina protocols.

### Tumor challenge

Four‐month‐old mice were fed 14 ppm eRapa for 3 months and challenged subcutaneously with 250,000 B16 cells as we described (Hurez *et al*., 2012; Hasty *et al*., 2014).

### Generation of bone marrow‐derived dendritic cells and *in vitro* Th‐cell differentiation

Bone marrow cells from tibias and femurs of 8‐ to 10‐week‐old BL6 mice were differentiated into dendritic cells (BMDC) with GM‐CSF (R&D Systems, Minneapolis, MN, USA) 4 ng/mL ± rapamycin (InvivoGen, San Diego, CA, USA) 5 ng/mL for 7 days. Sorted naïve T cells (CD4^+^CD25^−^) from 8‐week‐old BL6 mouse spleens were cocultured with matured BMDC for 5 days and tested for IL‐17A and IFN‐γ.

### Flagellin‐induced IL‐22

Two‐month‐old WT BL6 mice were given Eudragit or eRapa for 6 months. At age 8 months, mice were injected intravenously with 1 μg flagellin (InvivoGen) to induce IL‐22 as described (Kinnebrew *et al*., 2012). Mice were bled as indicated and serum assessed for IL‐22 by ELISA (eBioscience, San Diego, CA, USA).

### Metagenomic analysis

DNA processing and metagenomic analyses were by Second Genome (South San Francisco, CA, USA) by phylochip array, v.G3. PhyloChips were scanned on a genearray (Affymetrix, Santa Clara, CA, USA). Each scan was captured by Affymetrix software (GeneChip Microarray Analysis Suite). An Adonis test was used to find differences in the whole microbiome among discrete categorical or continuous variables.

### Bioinformatics

Comparisons of mean log‐expression of rapamycin‐treated samples vs controls were made with the standard limma package using Benjamini and Hochberg's correction to control the false discovery rate. Analyses were carried out for each cell type independently for each individual mouse (three mice per treatment group). Gene subsets related to specific biological functions were examined for change in each cell type and tabulated. Estimates of log‐ratio changes from LIMMA were submitted into Ingenuity Pathway Analysis (IPA; QIAGEN, Redwood City, CA, USA) and genespring (v.10; Agilent, Santa Clara, CA, USA) for analyses. Ingenuity Pathway Analysis was used to identify and visualize canonical networks consistently associated with eRapa across cell types using Core analysis and the Comparison tool.

### Statistics

Data are averages ± standard errors of the mean. Student's *t*‐test or analysis of variance was carried out as appropriate on graphpad prism 5 with *P *<* *0.05 significant. Survival curves were made by Kaplan–Meier method and compared by log‐rank test.

## Funding

No funding information provided.

## Conflict of interest

Under a licensing agreement between Rapamycin Holdings, Inc. and the University of Texas Health Science Center San Antonio, Z.D. Sharp, and P. Hasty, the university is entitled to milestone payments and royalty on sales of the rapamycin formulation used in this study. ZDS is an uncompensated member of the Rapamycin Holdings, Inc. scientific advisory committee.

## Author contributions

V.H., V.D., M.B., C.O., and T.C. designed research and analyzed the data; V.H., V.D., A.L., L.S., S.P., A.P., K.M., J.D., and Y.L. performed research; J.G. designed and performed statistical and bioinformatics analyses; V.G. provided animal husbandry and mouse cohorts; P.H. and Z.S. interpreted mTOR signaling data and provided reagents and intellectual contributions; V.H., V.D., and T.C. developed experimental approaches and wrote the manuscript.

## Supporting information


**Appendix S1** Supporting Material and Methods
**Fig. S1** Representative gating strategy for flow cytometry sorting.
**Fig. S2**–**S5** Representative gating strategy for flow cytometry analyses.
**Fig. S6** eRapa effects on T cells CD62L expression and *in vivo* migration.
**Fig. S7** eRapa alters T helper (Th) pathway differentiation (Ingenuity Pathway Analysis).
**Fig. S8** eRapa skews T‐cell differentiation in spleen and Peyer's patches.
**Fig. S9** eRapa alters myeloid and B‐cell subpopulation prevalence.
**Fig. S10** Rapamycin‐conditioned bone marrow‐derived dendritic cells skew naïve T cells toward a Th17 phenotype and how eRapa affects pro‐inflammatory factors in lungs.
**Fig. S11** eRapa affects innate lymphoid cells (ILCs) and gut microbial but does not protect against C. rodentium infection.
**Table S1** eRapa induces changes in genes regulating T‐cell homeostasis, markers of naïve versus memory or exhausted/senescent T cells and metabolism.
**Table S2** eRapa induces changes in chemokine and chemokine receptor genes in T cells.
**Table S3** eRapa induces changes in gene expression in B cells and myeloid cells genes regulating activation and differentiation.
**Table S4** eRapa induces changes in B‐cell gene expression.
**Table S5** eRapa induces changes in apoptosis‐ and inflammasome‐related gene expression.
**Table S6** eRapa induces changes in mTOR regulated and autophagy gene expression.

**Video S1** Activity of RAG2^‐/‐^ mice on eRapa at age 62 weeks (14.5 months).

**Video S2** Activity of RAG2^‐/‐^ mice on Eudragit control at age 58 weeks (13.5 months).
