# Supplementary material for: Chronic mTOR inhibition in mice with rapamycin alters T, B, myeloid, and innate lymphoid cells and gut flora and prolongs life of immune‐deficient mice
Source: Aging Cell. 2015 Aug 28;14(6):945–56. doi: 10.1111/acel.12380 (PMC4693453; doi:10.1111/acel.12380)
Supplement: Supplementary file 1 — Appendix S1 Supporting Material and Methods Fig. S1 Representative gating strategy for flow cytometry sorting. Fig. S2–S5 Representative gating strategy for flow cytometry analyses. Fig. S6 eRapa effects on T cells CD62L expression and in vivo migration. Fig. S7 eRapa alters T helper (Th) pathway differentiation (Ingenuity Pathway Analysis). Fig. S8 eRapa skews T‐cell differentiation in spleen and Peyer's patches. Fig. S9 eRapa alters myeloid and B‐cell subpopulation prevalence. Fig. S10 Rapamycin‐conditioned bone marrow‐derived dendritic cells skew naïve T cells toward a Th17 phenotype and how eRapa affects pro‐inflammatory factors in lungs. Fig. S11 eRapa affects innate lymphoid cells (ILCs) and gut microbial but does not protect against C. rodentium infection. Table S1 eRapa induces changes in genes regulating T‐cell homeostasis, markers of naïve versus memory or exhausted/senescent T cells and metabolism. Table S2 eRapa induces changes in chemokine and chemokine receptor genes in T cells. Table S3 eRapa induces changes in gene expression in B cells and myeloid cells genes regulating activation and differentiation. Table S4 eRapa induces changes in B‐cell gene expression. Table S5 eRapa induces changes in apoptosis‐ and inflammasome‐related gene expression. Table S6 eRapa induces changes in mTOR regulated and autophagy gene expression. [file ACEL-14-0945-s001.pdf]

Supporting Information

Supporting Experimental Procedures

Flow cytometry. We isolated and stained cells for surface markers or intracellular cytokines, and perform cell sorts as previously described (Curiel *et al.* 2003). Data were acquired on a LSRII flow cytometer (BD Biosciences) and analyzed using FlowJo software (Tree Star Inc., Ashland, OR). Anti-CD11b (M1/70), anti-CD11c (HL3), anti-mouse IgM (Il/41), anti-interferon (IFN)-γ (XMG1.2), anti-CD25 (PC61), anti-CD69 (H1.2F3), anti-CD4 (GK1.5), anti-CD3 (500A2), anti-Gr-1 (RB6-8C5), anti-PD-1 (J43), anti-CD23 (B3B4), anti-IL-4 (11B11), anti-IL-12 (C15.6), anti-NK1.1 (PK136) and matched isotype control antibodies were from BD Biosciences. Anti-CD62L (MEL14), anti-MHC Class II (I-A/I-E) (M5/114.15.2), anti-CD45R (B220) (RA3-6B2), anti-CD93 (AA4.1), anti-CCR4 (2G12), anti-IL-6 (MP5-20F3), anti-IL10 (JES5-16E3), anti-IL-17A (17B7), anti-IL-22 (IL22J0P), anti-LAG3 (C9B7W) and anti-Foxp3 (FJK-16a) were purchased from eBioscience. Anti-CD8α (5H10) was from Caltag Laboratories, anti-CD80 (16-10A1), anti-NKp46 (CD335, 29A.1.4), and anti-cKit (CD117, 2B8) from Biolegend. Anti-CXCR3 (Clone 220803), anti-CCR6 (Clone 140706) were from R&D Systems. Purified Fc receptor blocking antibody CD16/32, functional grade anti-mouse CD3 (145-2C11) and CD28 (37.51) were purchased from eBioscience (San Diego, CA).

Whole genome gene expression. Spleen cells from 22-month old male mice fed eRapa or Eudragit for 12 months were sorted to high purity using a BD FACSAria flow cytometer (BD Biosciences) in RNAse-free phosphate buffered saline (PBS) + 2% bovine serum albumin. Total cellular RNA was purified using a Quick-RNA MicroPrep kit (Zymo Research). RNA quality was assessed using an Agilent Eukaryotic Total RNA Nano assay. cRNA probes preparation and hybridization to Illumina MouseWG-6 Version 2 BeadChips was conducted at the UTHSCSA Genomics Core Facility using

standard Illumina protocols. Gene expression data was normalized (quantile normalization with background subtraction) using Illumina GenomeStudio software version 1.9.0.

Treg suppression. 3 x 10^4 flow cytometry-purified $CD4^+CD25^-$ T cells (effector T cells) from naïve C57/BL6 mice were incubated for ~9 minutes at 37° C in 10 μM carboxy fluorescein succinimidyl ester (CFSE) (Invitrogen) in phosphate buffered saline and re-suspended in medium RPMI-1640. Sorted $CD4^+CD25^{hi}$ T cells (Tregs) were added in indicated ratios together with Dynabeads Mouse CD3/CD28 T Cell Expander (Invitrogen) beads at 1 bead:3 effector T cell. After 72 hours, percentage of Treg suppression was calculated as 100- [(effector T cell proliferation without Treg – effector T cell with Treg)/effector T cell without Treg] x 100%.

***In vivo* T cell trafficking**. T cells were purified from spleens of eRapa or Eudragit treated mice using a mouse T lymphocyte enrichment set-DM (BD Biosciences), labeled them with 1 μM (eRapa, $CFSE^{low}$) or 10 μM (Eudragit, $CFSE^{hi}$) CFSE and 10x10^6 T cells were injected intravenously at a 1:1 ratio into 3 young BL6 recipients. The next day, proportions of $CFSE^{low}$ versus $CFSE^{hi}$ T cells were assessed by flow cytometry in various organs, after making single-cell organ suspensions.

Generation of bone marrow-derived dendritic cells and *in vitro* Th cell differentiation. Bone marrow cells from tibias and femurs of 8-10 week old BL6 mice were collected as we described(Hurez *et al.* 2012) and differentiated into bone marrow-derived dendritic cells (BMDC) using GM-CSF (R&D Systems) at 4 ng/ml ± rapamycin (InvivoGen) at 5 ng/ml for 7 days. BMDCs were identified as $CD11b^+CD11c^+$ cells by flow cytometry and then matured with either *E. coli* LPS (InvivoGen) at 1 μg/ml or IL-1β/TNF-α (R&D Systems) at 10 ng/ml each for 2 days. For T helper cell differentiation, sorted naive T cells ($CD4^+CD25^-$) from 8 week old BL6 mouse spleens were added to matured BMDCs at a T:BMDC ratio of 5:1 with anti-CD3 and anti-CD28 antibodies (both at 2 μg/ml and from BD Biosciences) in RPMI-1640 medium plus 10% fetal calf serum, 4 mM glutamine, 10 mM

HEPES and antibiotics (Gibco). After 5 days, cells were harvested and incubated in Leukocyte Activation Cocktail (BD Biosciences) for 5 hours. Surface staining was done with anti-CD4 and anti-CD3 antibodies. For subsequent intracellular cytokine staining, cells were fixed and permeabilized with the Foxp3 fix/perm buffer (eBioscience), then stained with anti-IL-17A and anti-IFN-γ antibodies.

***Citrobacter rodentium* infection**. 2 month old RAG2$^{-/-}$ mice (BL6 background) were started on Eudragit or eRapa for 1 month. At 3 months of age, mice were infected with 1 x 10^9 colony forming units of *C. rodentium* (strain DBS100, generously provided by Dr. John Leong, Tufts University) in 200 μl PBS by oral gavage as described(Basu *et al.* 2012). Body weights were assessed on a daily basis. Mice were sacrificed when body weight dropped below 20% of initial weight.

Necrosis and apoptosis induction. Total spleen cells were freeze-thawed for 3 cycles of -20° C. freezing followed by 37° C. thawing to induce necrosis, or were incubated overnight at 37° C. in RPMI without fetal bovine serum to induce apoptosis. Apoptosis was assessed using a Vybrant Apoptosis Assay kit (Invitrogen) following the manufacturer's protocol. Culture supernatants from these apoptotic or necrotic cell cultures were used to stimulate spleen cells from young, naïve BL6 mice for 48 hours *in vitro* before measurement of intracellular cytokines by flow cytometry as described above.

A20 and IkB-α expression lungs. Western blots from tissue lysates were prepared from lungs of 22 month UM-HET3 mice that had been fed low (4.7 ppm)-, mid (14 ppm)-, and high (42 ppm)-dose eRapa or control diet for 13 months from 9 to 22 months of age (n=6/cohort). Anti-A20 and anti-IκB-α antibodies were from Santa Cruz Biotechnology. Anti-actin antibody was from Bethyl laboratories. Actin was probed on the same membranes and used as a loading control.

Metagenomic analysis. DNA isolates were prepared from murine feces using a Mo-Bio PowerSoil DNA isolation kit following the manufacturer's protocol. DNA sample processing and metagenomic

analyses were performed by Second Genome (South San Francisco, CA). Briefly, bacterial 16S rRNA genes were amplified using the degenerate forward primer: 27F.1 5'-AGRGTTTGATCMTGGCTCAG-3' and the non degenerate reverse primer: 1492R.jgi 5'-GGTTACCTTGTTACGACTT-3'. Samples were quantified by electrophoresis using an Agilent 2100 Bioanalyzer. Labeled bacterial products were fragmented, biotin labeled, and hybridized to a PhyloChip Array, version G3. PhyloChip arrays were scanned using a GeneArray scanner (Affymetrix). Each scan was captured using standard Affymetrix software (GeneChip Microarray Analysis Suite). Hybridization values, the fluorescence intensity for each taxon, were calculated as a trimmed average, with maximum and minimum values removed before averaging. An Adonis test was used for finding significant differences in the whole microbiome among discrete categorical (age, diet) or continuous (proportions of immune cell populations) variables. The samples were randomly reassigned to the various sample categories, and the fraction of permutations with larger cross-category differences relative to within-category differences is reported as the p value for the Adonis test.

Bioinformatic analyses. The comparisons of the mean log-expression of rapamycin treated samples versus the control samples were performed using the LIMMA package(Smyth 2004). Analyses were conducted for each of the cell types independently. Subsets of genes related to specific biological functions (exhaustion/senescence, cytokine signaling, chemokines, apoptosis, DNA damage, metabolic pathways, and co-signaling) were examined for change due to eRapa in each cell type and tabulated. The estimates of the log-ratio changes in expression from the LIMMA contrasts by cell type were submitted into Ingenuity Pathway Analysis (IPA,QIAGEN Redwood City, CA) and Genespring (version 10, Agilent Technologies) for network and pathway analyses. Ingenuity Pathway Analysis was used to identify and visualize canonical networks that were consistently associated with rapamycin treatment across cell types using the Core analysis and the Comparison tool.

Statistical analysis. Data are expressed as averages ± standard errors of the mean. Student's *t* test or analysis of variance was performed as appropriate using GraphPad Prism 5 with $p<0.05$ considered significant. Survival curves were generated by the Kaplan-Meier method and compared with a log-rank statistic.

References

Basu R, O'Quinn DB, Silberger DJ, Schoeb TR, Fouser L, Ouyang W, Hatton RD, Weaver CT (2012). Th22 cells are an important source of IL-22 for host protection against enteropathogenic bacteria. *Immunity*. **37**, 1061-1075.

Curiel TJ, Wei S, Dong H, Alvarez X, Cheng P, Mottram P, Krzysiek R, Knutson KL, Daniel B, Zimmermann MC, David O, Burow M, Gordon A, Dhurandhar N, Myers L, Berggren R, Hemminki A, Alvarez RD, Emilie D, Curiel DT, Chen L, Zou W (2003). Blockade of B7-H1 improves myeloid dendritic cell-mediated antitumor immunity. *Nat Med*. **9**, 562-567.

Hurez V, Daniel BJ, Sun L, Liu AJ, Ludwig SM, Kious MJ, Thibodeaux SR, Pandeswara S, Murthy K, Livi CB, Wall S, Brumlik MJ, Shin T, Zhang B, Curiel TJ (2012). Mitigating age-related immune dysfunction heightens the efficacy of tumor immunotherapy in aged mice. *Cancer Res*. **72**, 2089-2099.

Smyth GK (2004). Linear models and empirical bayes methods for assessing differential expression in microarray experiments. *Stat Appl Genet Mol Biol*. **3**, Article3.

Figure S1

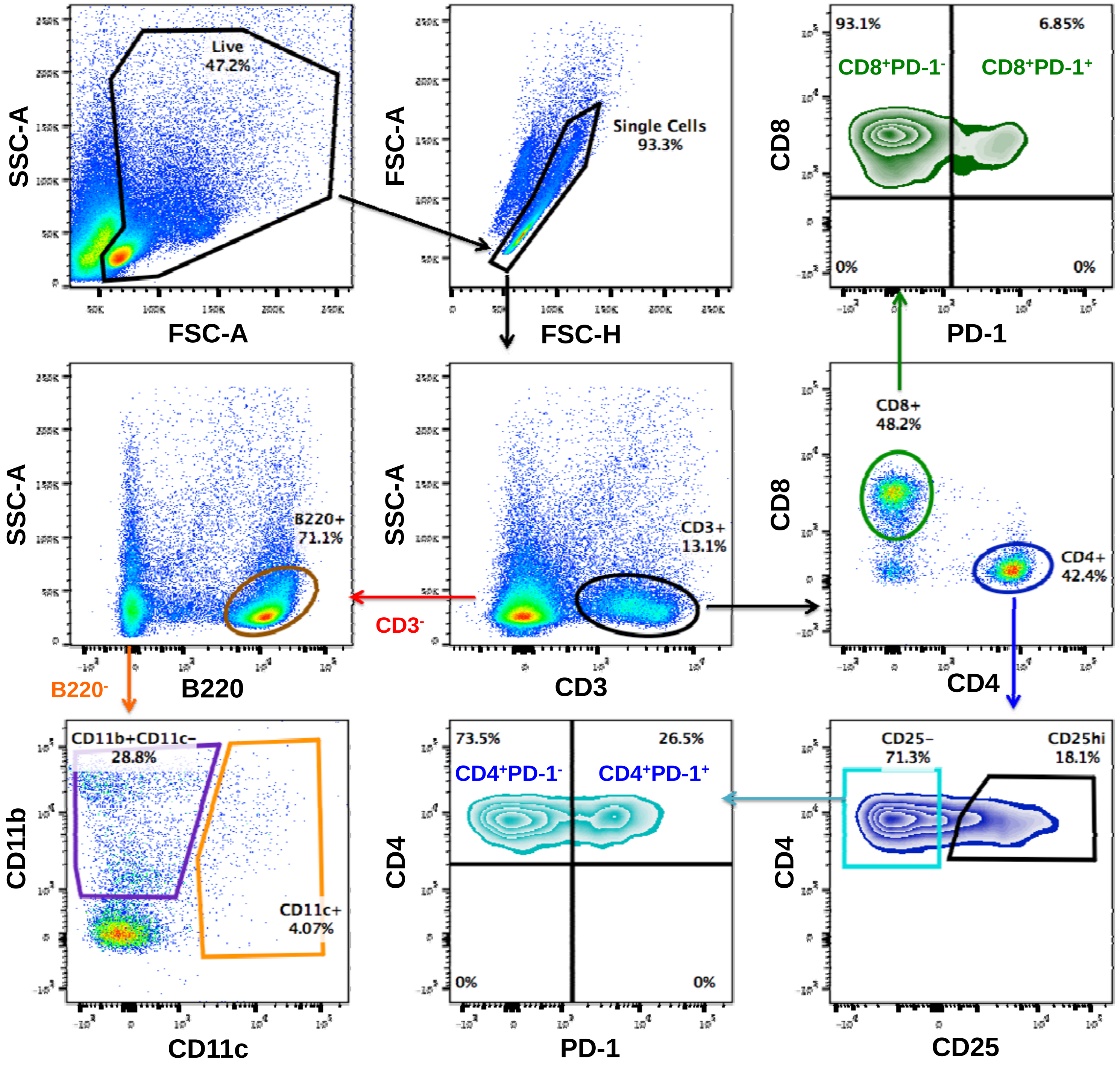

Fig. S1 Representative flow cytometry plots showing gating strategy for sorting immune cell populations from spleen of an aged male Bl6 mouse (1 out of 3 on eRAPA) used to analyze gene expression by microarrays.

Figure S2

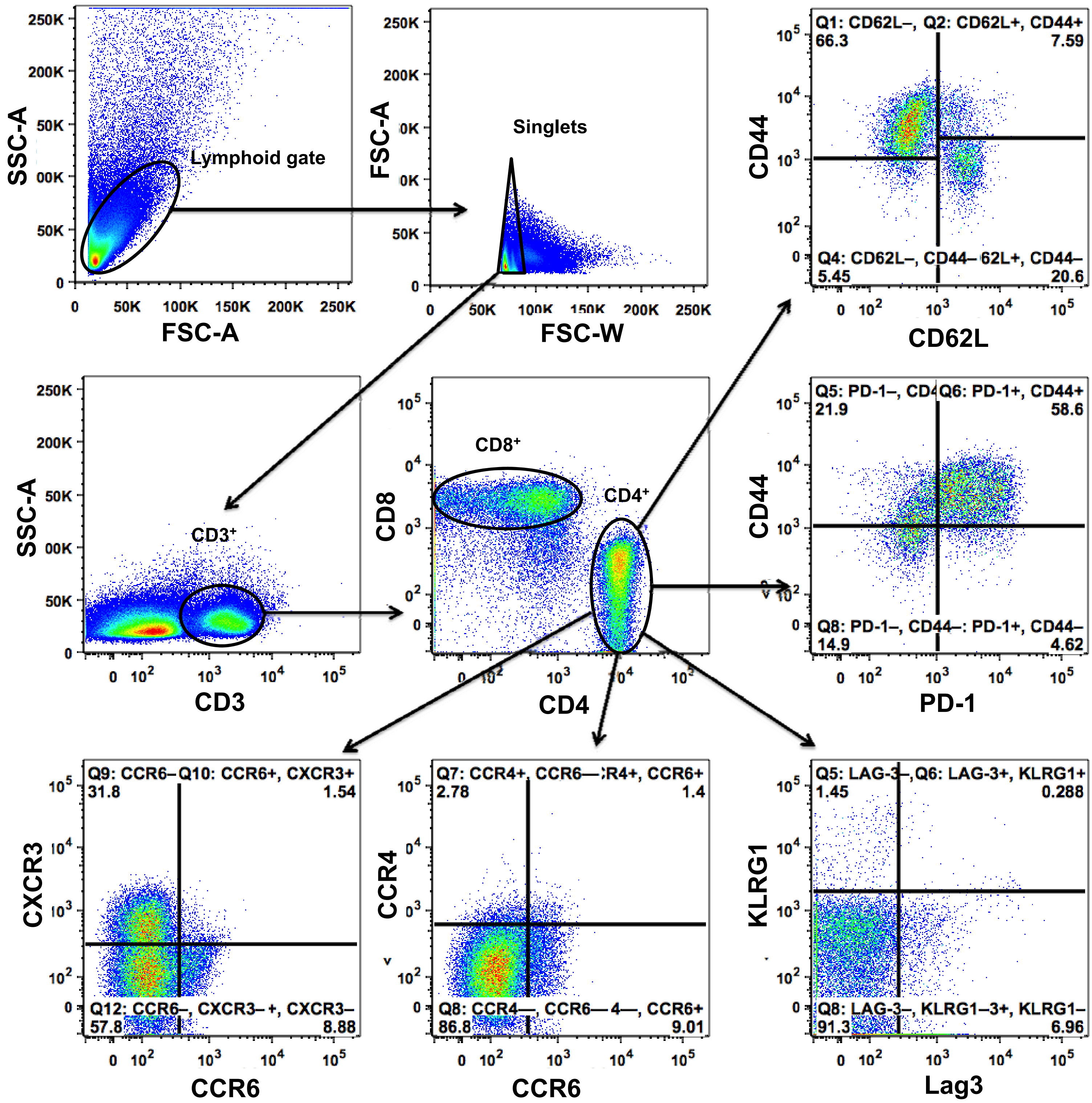

Fig. S2 Representative flow cytometry plots showing gating strategy for the analyses of memory/exhaustion markers (CD44, CD62L, PD-1, Lag3, KLRG1) and chemokine receptors (CXCR3, CCR6, CCR4) in $CD4^+$ T cells from spleen of an aged Bl6 mouse. Similar gating was used to analyze expression in $CD8^+$ T cells.

Figure S3

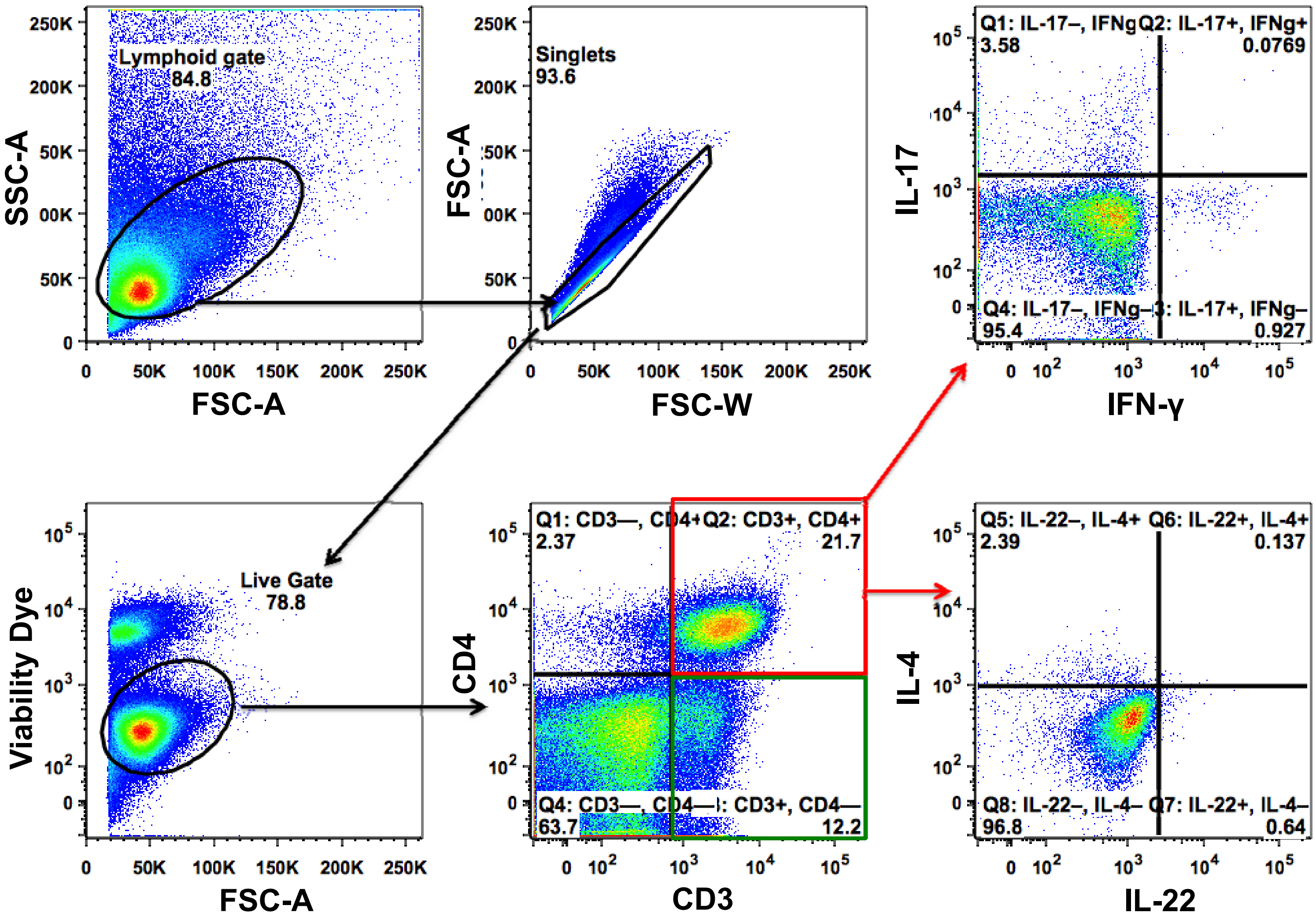

Fig. S3 Representative flow cytometry plots showing gating strategy for the analyses of intracellular cytokines (IFNg,IL-17A, IL-4, IL-22) in $CD4^+$ T cells (red box, $CD3^+$ $CD4^+$) from spleen of an aged Bl6 mouse. Similar gating was used to analyze expression in $CD8^+$ T cells (green box, $CD3^+$ $CD4^-$) and in $CD3^-$ $CD4^+$ “LTI-like” cells.

Figure S4

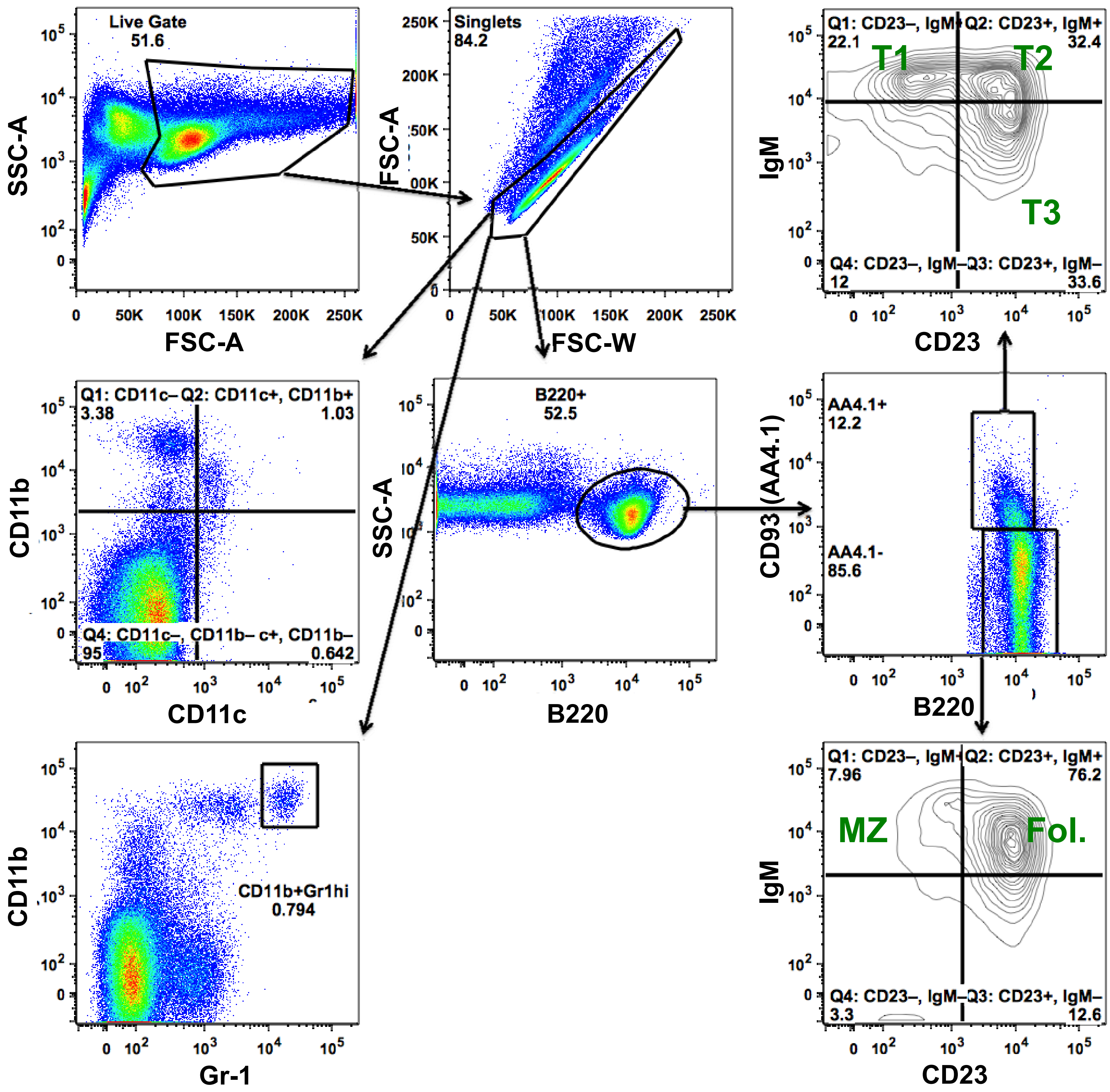

Fig. S4 Representative flow cytometry plots showing gating strategy for the analyses of $CD11b^{+}CD11c^{-}$ and $CD11b^{+}Gr\text{-}1^{hi}$ myeloid cells, $CD11c^{+}$ dendritic cells and B cell subsets from spleen of an aged Bl6 mouse. T1, T2, T3 = transitional B cells, MZ = marginal zone B cells, Fol. = follicular B cells

Figure S5

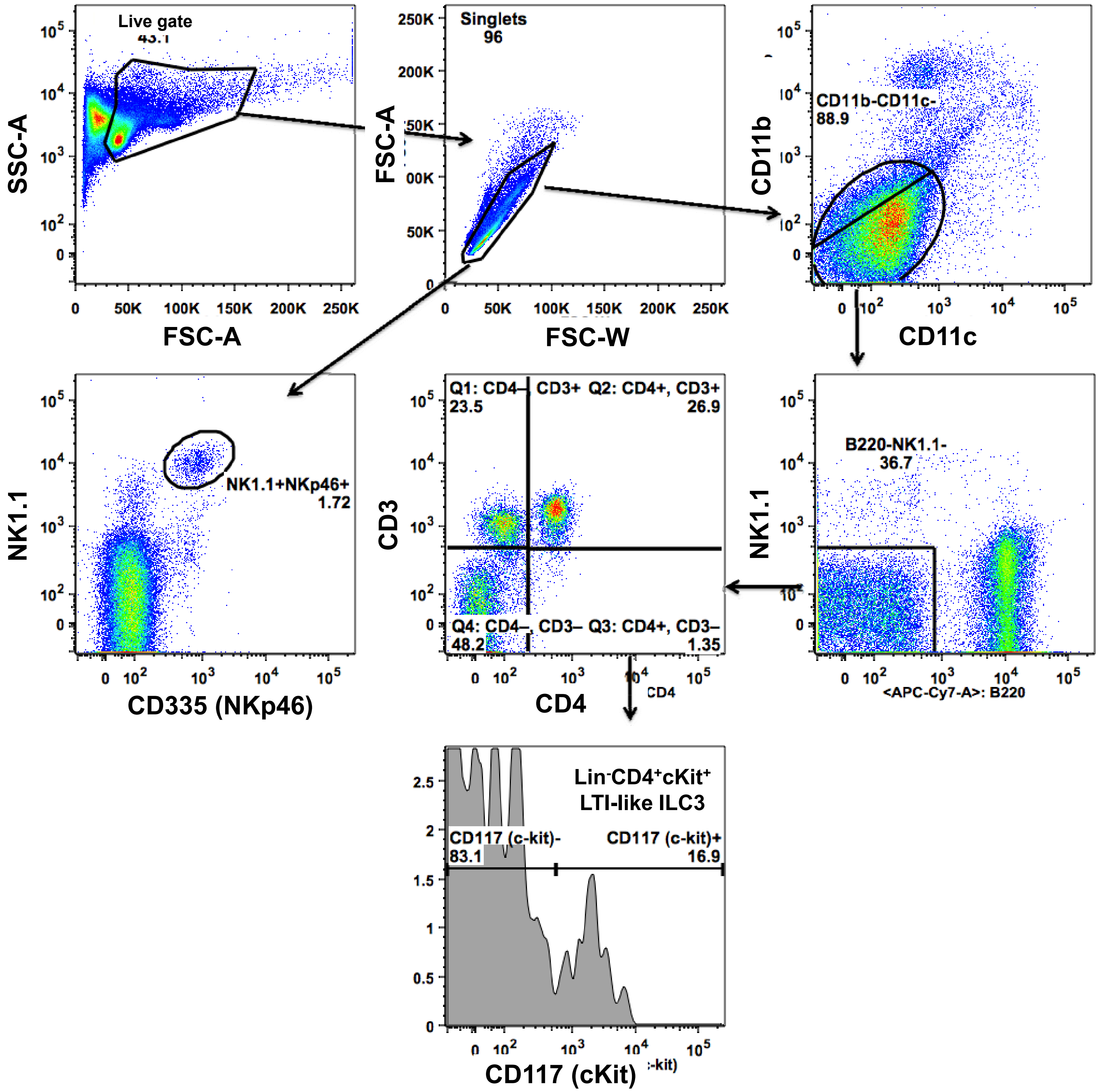

Fig. S5 Representative flow cytometry plots showing gating strategy for the analyses of NK cells and Lin-CD4+cKit+ LTI-like ILC3 cells.

Figure S6

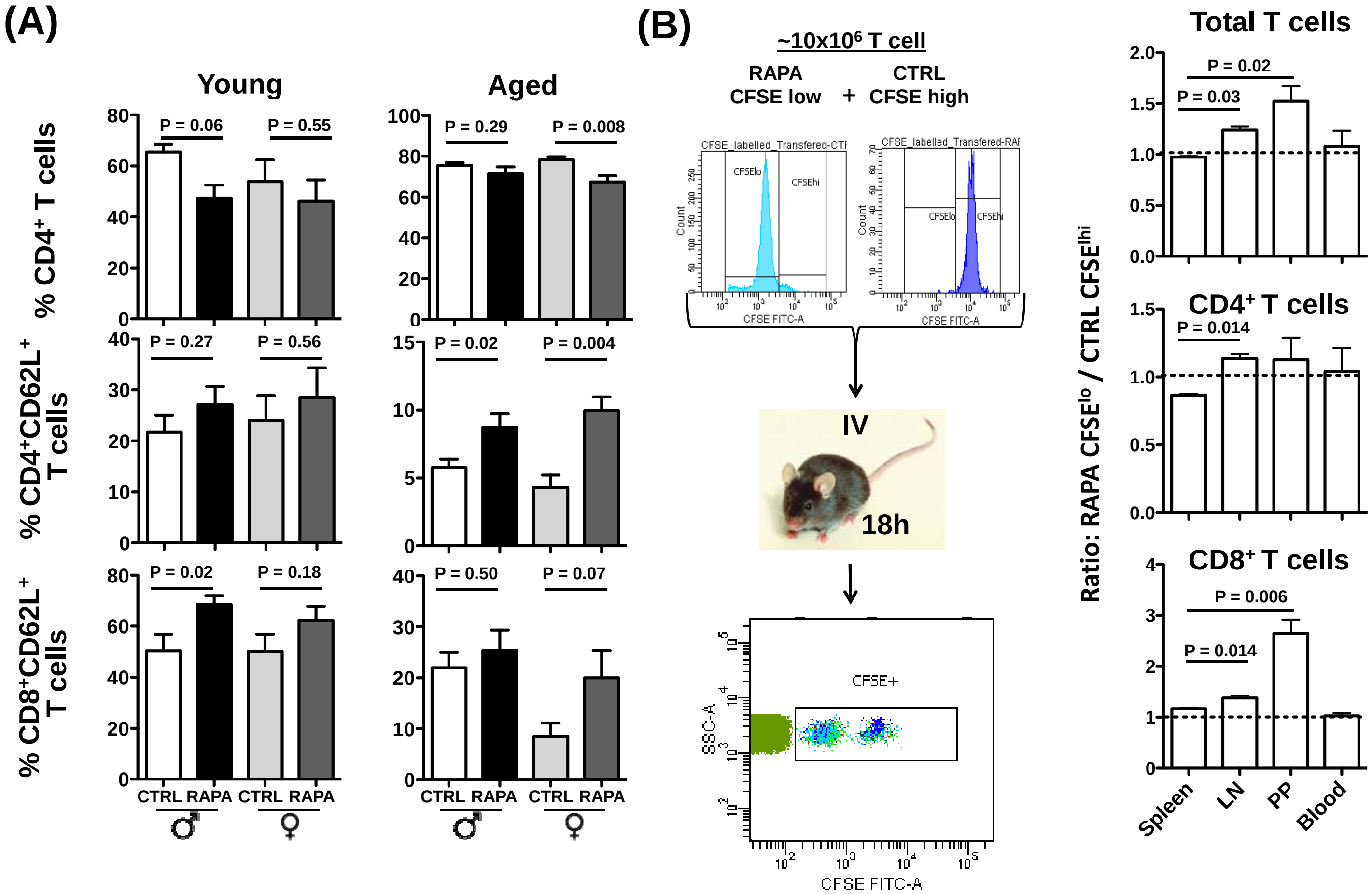

Fig. S6 eRapa affects T cells CD62L expression and *in vivo* migration. (*A*) Frequency of $CD4^+$ T cells and $CD62L^+$ cells in $CD4^+$ and $CD8^+$ T cells from Peyer's patches of young (8 month old) and aged (24-25 month old) male and female C57BL/6 mice on Eudragit (CTRL) or eRapa (RAPA) chow for 6 months. (n = 10-15 mice/group). (*B*) Spleen T cells from eRapa or Eudragit (CTRL) treated mice were isolated, labeled with low (eRapa) or high (CTRL) CFSE dye, mixed at 1:1 ratio and injected intravenously into untreated young recipients. After 18 hours, proportions of migrated cells in various organs were analyzed by flow cytometry. Histograms indicate the ratio "proportion of T cells from eRapa mice/proportion of T cells from CTRL mice" in spleen, lymph nodes (LN), Peyer's patches (PP) and peripheral blood (n = 3 recipient mice). All error bars represent standard error of mean (S.E.M.)

Figure S7

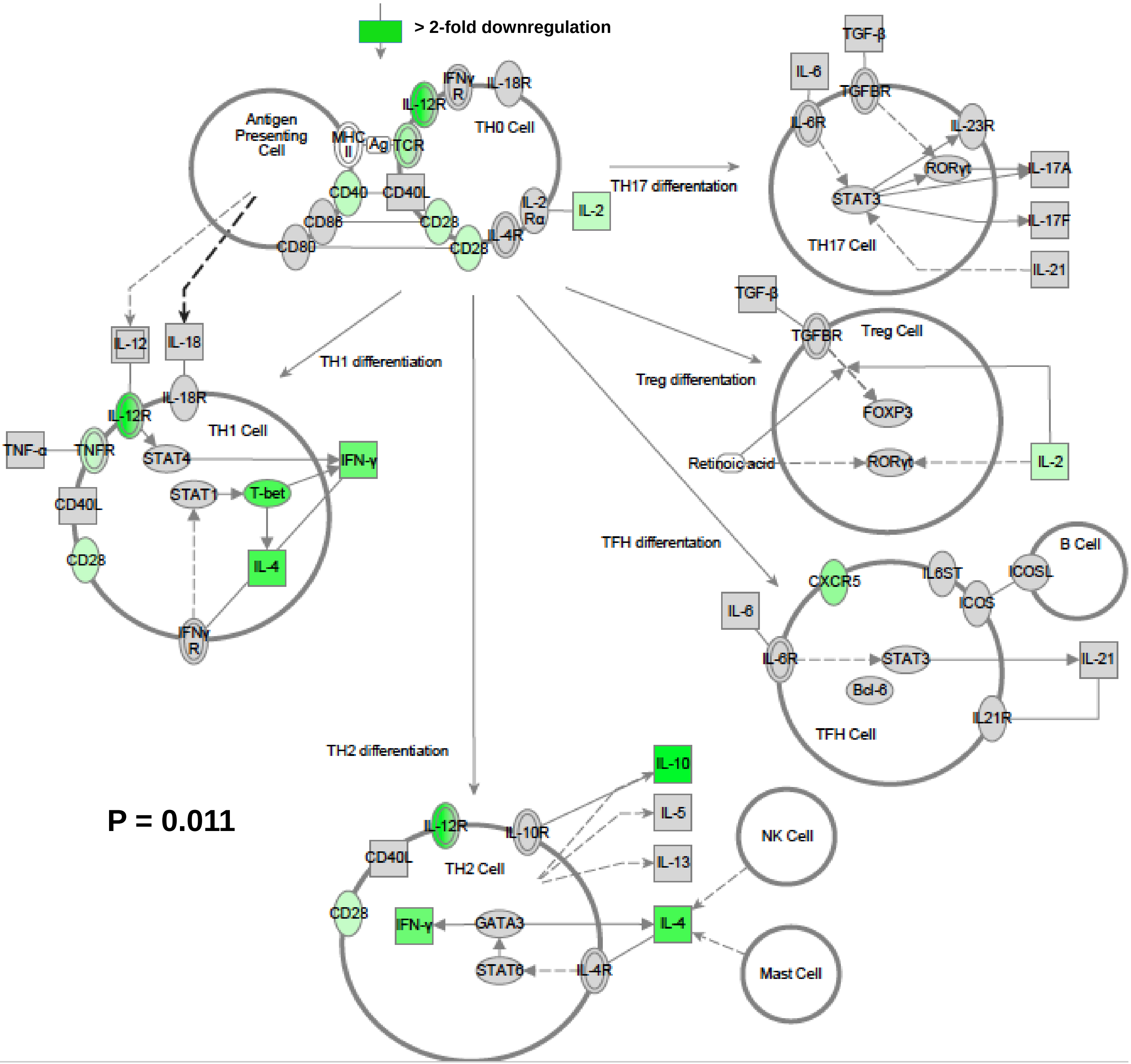

Fig. S7. eRapa alters T helper (Th) pathway differentiation. Th differentiation pathway (Ingenuity Pathway Analysis) showing downregulated gene expression (green) of cytokines and transcription factors in CD4$^+$PD-1$^-$ T cells from eRapa- treated versus Eudragit control mice. P value from IPA statistical analysis.

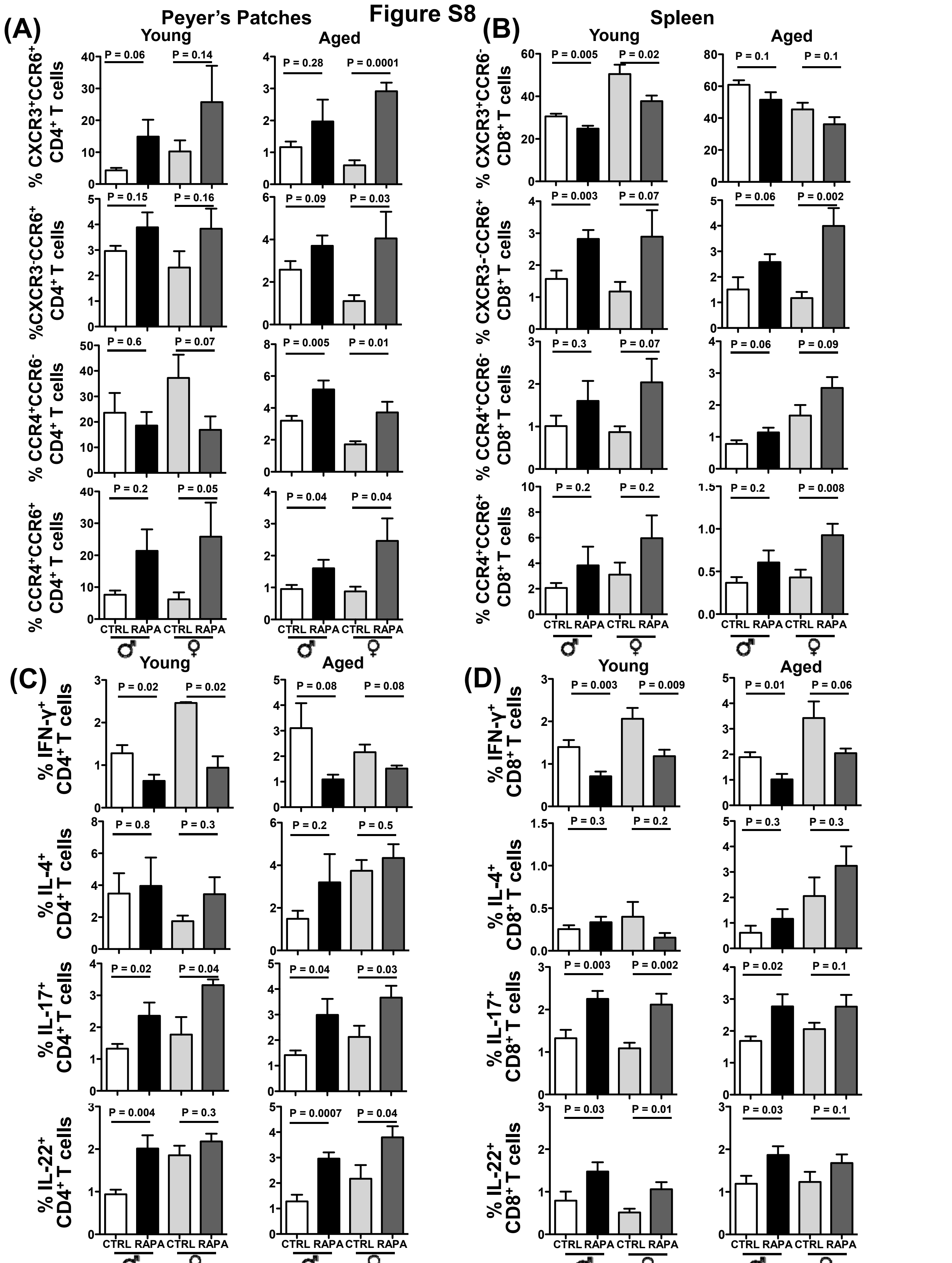

Fig. S8 eRapa skews T cell differentiation in spleen and Peyer's patches. (*A*) Frequency of chemokine receptor expressing cells in CD4$^+$ T cells from Peyer's patches of young (8 month old) and aged (24-25 month old) males and females C57BL/6 mice on Eudragit (CTRL) or eRapa (RAPA) chow for 6 months. (n = 10-15 mice/group). (*B*) Chemokine receptor expressing cell prevalence in CD8$^+$ splenic T cells from young (8 months) and aged (24-25 months) male and female C57BL/6 mice on Eudragit (CTRL) or eRapa (RAPA) chow for 6 months. (n = 10-15 mice/group). (*C*) Frequency of cytokine expressing cells in CD4$^+$ T cells from Peyer's patches of young (8 month old) and aged (24-25 month old) male and female mice on Eudragit (CTRL) or eRapa (RAPA) chow for 6 months. (n = 10-15 mice/group). Cytokines detected by intracellular flow cytometry after 18 hour stimulation with anti-CD3/CD28 beads. (*D*) Cytokine expressing cell prevalence in CD8$^+$ splenic T cells. Cytokines detected by intracellular flow cytometry after 18 hour stimulation with anti-CD3/CD28 beads. All error bars represent S.E.M.

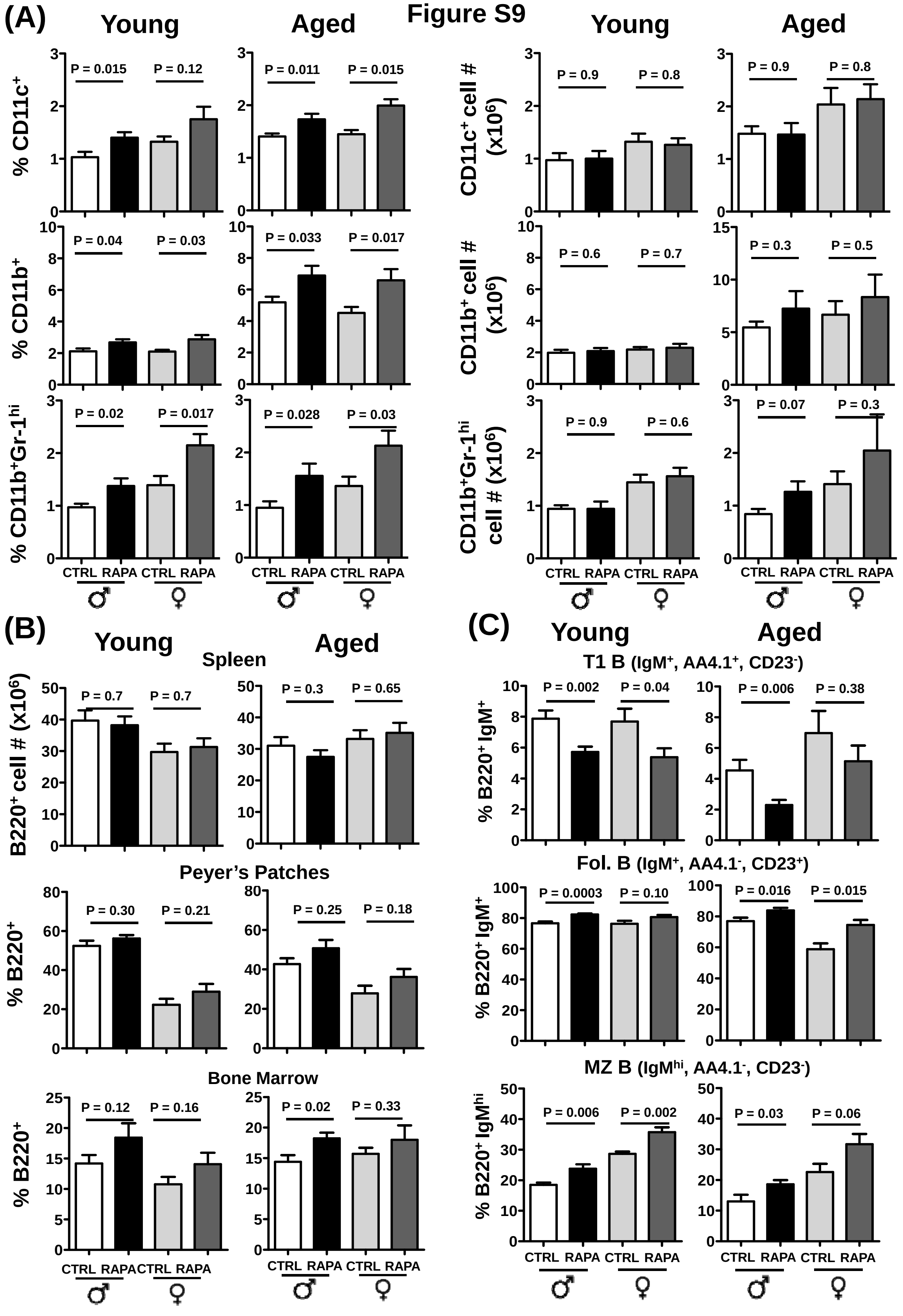

Fig. S9. eRapa alters myeloid and B cell subpopulation prevalence. (*A*) Prevalence and numbers of $CD11b^+CD11c^+$ dendritic cells, $CD11b^+CD11c^-$ monocytes/macrophages and $CD11b^+Gr\text{-}1^{hi}$ cells from spleens of young (8 month old) and aged (24-25 month old) males and females C57BL/6 mice on Eudragit (CTRL) or eRapa (RAPA) chow for 6 months. (n = 10-15 mice/group). (*B*) Number of $B220^+$ B cells in spleens, and their prevalence in Peyer's patches and bone marrows of young (8 month old) and aged (24-25 month old) male and female C57BL/6 mice on Eudragit (CTRL) or eRapa (RAPA) chow for 6 months (n = 10-15 mice/group). (*C*) Frequency of transitional (T1 B), follicular (Fol. B) and marginal zone (MZ B) B cells in spleens of young (8 month old) and aged (24-25 month old) male and female C57BL/6 mice on Eudragit (CTRL) or eRapa (RAPA) chow for 6 months (n = 10-15 mice/group).

Figure S10

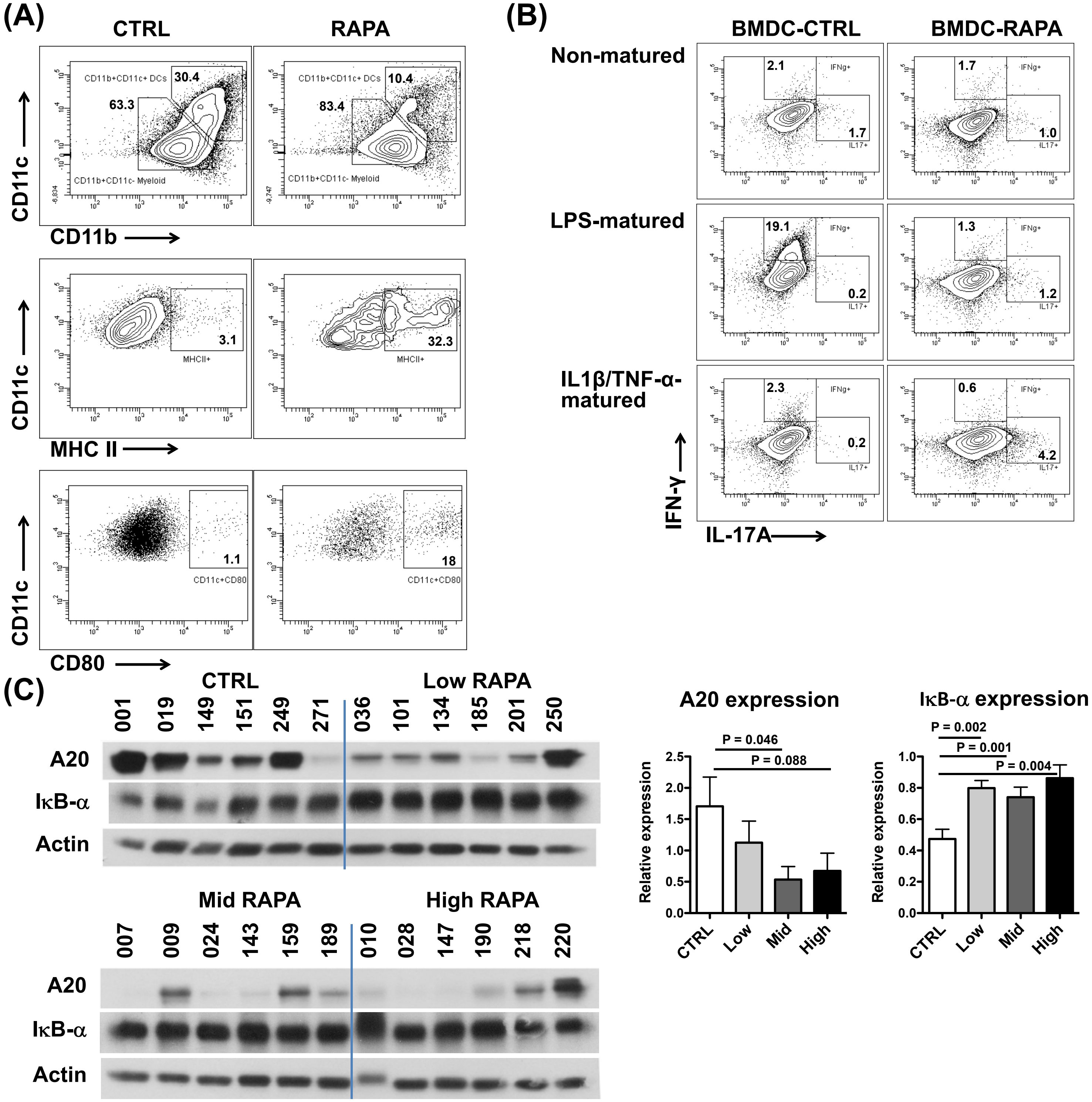

Fig. S10 Rapamycin conditioned bone marrow derived dendritic cells (BMDC) skew naïve T cells toward Th17 and affect pro-inflammatory factors in lungs. (*A*) Bone marrow derived dendritic cells (BMDC) differentiated in rapamycin (RAPA). Bone marrow cells were cultured with either GM-CSF/DMSO or GM-CSF/rapamycin at 5 ng/ml for 7 days to generate BMDCs. Flow cytometry was used to define BMDCs as $CD11b^+CD11c^+$ (top) and activated BMDCs were defined as $CD11c^+MHCII^+$ or $CD11c^+CD80^+$. GM-CSF, granulocyte macrophage-colony stimulating factor. DMSO, dimethylsulfoxide vehicle control. MHC, major histocompatibility complex. (*B*) $CD4^+CD25^-$ cells from 12 week old female BL6 mice co-cultured with BMDCs generated with either GM-CSF/DMSO (CTRL) or GM-CSF/rapamycin (RAPA) and matured with LPS or cytokines (IL-1β + TNF-α). After 5 days, IL-17 and IFN-γ was assessed by intracellular cytokine stain in $CD4^+$ T cells using flow cytometry. GM-CSF, granulocyte macrophage-colony stimulating factor. DMSO, dimethylsulfoxide. TNF-α, tumor necrosis factor-α. Numbers in quadrants are percent gated events. One representative experiment of two. (*C*) Effects of eRapa diet on the expression of A20 and IκB-α in lungs. Immunoblots and densitometric analysis in tissue lysates prepared from lungs of 22 month old UM-HET3 mice that had received low- (4.7 ppm), mid- (14 ppm), or high (42 ppm)-dose eRapa or control diet for 13 months from 9 to 22 months of age (n=6/cohort). Antibodies used displayed on the left. Actin was probed on the same membranes and used as a loading control. Histograms show the ratio (protein/actin) of intensity values. P values from unpaired *t* test. Mouse numbers at gel tops. ppm, parts per million. All error bars represent S.E.M

Figure S11

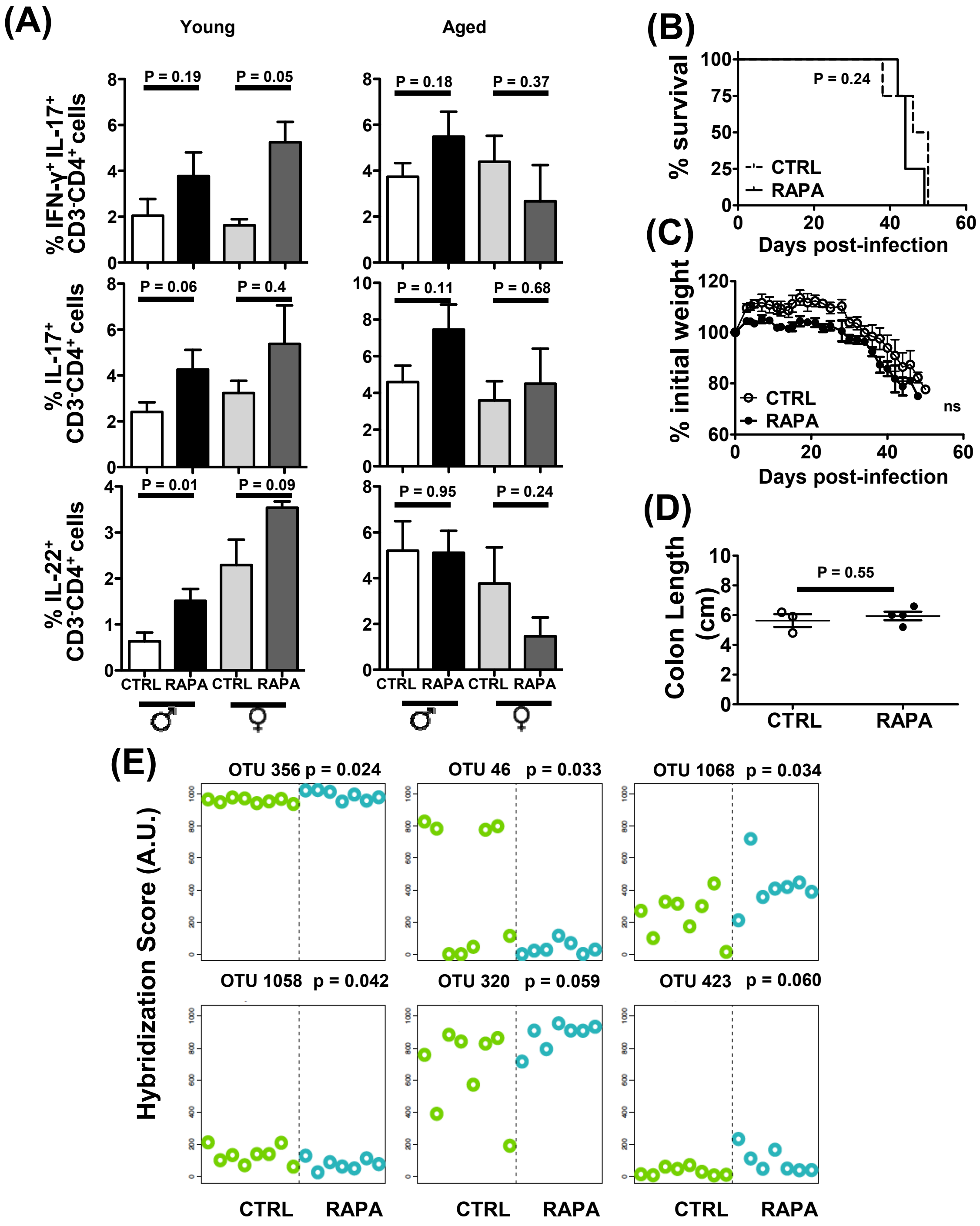

Taxonomic annotations of the OTUs with the lowest p-values

Taxa No	Phylum	Class	Order	Family	Genus	Species
46	Firmicutes	Bacilli	Lactobacillales	Lactobacillaceae	Lactobacillus	Lactobacillus intestinalis
356	Acidobacteria	Acidobacteria	Acidobacteriales	Acidobacteriaceae	unclassified	unclassified
1056	Bacteroidetes	Bacteroidia	Bacteroidales	Rikenellaceaell	unclassified	unclassified
1068	Bacteroidetes	Bacteroidia	Bacteroidales	Rikenellaceaell	unclassified	unclassified

Fig. S11 eRapa affects ILCs and gut microbial but does not protect against *C. rodentium* infection. (*A*) eRapa increases Peyer's patch ILCs IL-17 and IL-22. Cytokine expressing cell prevalence in $CD3^-CD4^+$ LTI-like cells from Peyer's patches of young (8 month old) and aged (24-25 month old) males and females mice on Eudragit (CTRL) or eRapa (RAPA) chow for 6 months (n = 10-15 mice/group). Cytokines detected by intracellular flow cytometry after 18 hour stimulation with anti-CD3/CD28 beads. (*B*) Survival of $RAG2^{-/-}$ BL6 mice on Eudragit (CTRL) or eRapa (RAPA) after infection with $1x10^9$ colony forming units of *C. rodentium* (N=4/group). P value, log-rank test. RAG2, recombinase activating gene 2. (*C*) Weight and (*D*) colon length of *C. rodentium* infected $RAG2^{-/-}$. P values, 2-way ANOVA and unpaired *t* test, respectively. (*E*) Effect of eRapa on gut metagenomes. Top, selected gut flora operational taxonomic units (OTUs). Hybridization scores from PhyloChip. OTU number and P values (unpaired *t* test,) indicated. Bottom, taxonomic classification of the OTUs with most significant difference between Eudragit (CTRL) and eRapa (RAPA) fed aged male and female mice (n = 7 mice/group). A.U., arbitrary units.

Supporting Tables

Table S1. eRapa induces changes in genes regulating T cell homeostasis, markers of naïve versus memory or exhausted/senescent T cells and metabolism.

GENES	CD4+ PD1-	CD4+ PD1+	CD8+ PD1-	CD8+ PD1+
BATF	-0.53	-0.36	0.27	-0.21
BCL2	0.58	0.49	0.18	0.12
BTLA	0.13	-0.02	-0.81	0.46
CCR5	-5.49	-3.21	-0.75	-1.98
CCR7	0.97	1.40	1.05	1.90
CD2	-0.11	0.03	0.05	-0.05
CD27	0.09	0.01	0.49	0.09
CD28	-1.00	-0.31	0.25	-0.93
CD3E	0.02	-0.06	0.24	0.00
CD44	-1.40	-0.08	-0.24	-0.71
CD48	-0.06	-1.11	-0.34	-0.96
CD69	-0.24	-0.08	-0.37	0.05
CD160	-1.21	-0.43	-0.37	-0.32
CD244 (2B4)	0.37	0.12	-1.85	-1.40
CTLA4	-1.19	0.07	0.31	0.34
FASL	-1.09	-1.06	-0.55	-1.34
FCGR3 (CD16)	-0.07	-4.14	-0.97	-3.28
HAVCR2 (Tim-3)	0.00	-0.95	-1.43	-2.05
ICOS	-0.04	0.53	0.19	0.52
IL2	-1.09	0.47	-0.21	0.32
IL2RA (CD25)	-0.37	0.33	0.43	-0.55
IL2RB (CD122)	-0.77	-0.27	0.16	-0.24
IL7R	0.38	0.38	0.75	0.38
ITGA3 (CD49c)	0.00	-0.66	0.00	0.56
ITGA4 (CD49d)	-0.43	-0.44	-0.01	-0.13
ITGAL (CD11a)	-0.91	-0.78	-0.39	-0.96
ITGAM (CD11b)	0.00	1.37	0.00	0.00
ITGB1 (CD29)	-0.42	-0.33	-0.62	0.12
KLF2	0.72	-0.03	0.18	0.29
KLRB1A	1.63	0.21	-0.72	2.86
KLRB1B	0.61	-0.97	0.51	-1.05
KLRB1C	-1.43	0.00	0.16	-0.01
KLRB1F	-2.25	-1.82	-0.70	-2.69
KLRD1 (CD94)	0.41	-0.40	0.59	0.61
KLRG1	-5.18	0.20	-1.25	-3.43
LAG3	-0.92	-1.09	-0.02	-0.86
LCP2 (SLP-76)	-0.02	0.02	0.08	0.10
LY6A (Sca-1)	-0.53	-0.78	-1.16	-0.16
MKI67 (Ki67)	-2.13	-2.07	-1.70	-3.90
NCAM1 (CD56)	0.76	0.97	0.23	0.21
NFATC1	-0.47	-0.50	-0.19	-0.28
OPTN	-2.15	-3.32	0.00	-2.51
PDCD1 (PD-1)	1.61	-0.95	0.13	-0.13
PRDM1 (Blimp-1)	-1.70	-1.77	-1.61	-0.53
PTPRC (CD45)	-0.75	-0.63	-0.19	-0.53
SELL (CD62L)	0.79	0.94	0.36	0.85
SLAM	-2.26	-3.15	-1.89	0.00
TNFSF10 (TRAIL)	0.00	-1.91	0.38	-1.66

	Gene	CD4+ PD1-	CD4+ PD1+	CD8+ PD1-	CD8+ PD1+
Fatty acids biosynthesis	ACACB	-0.99	-0.73	1.50	0.94
	ACSL1	0.02	0.91	0.05	0.21
	FAS	-0.37	-0.07	0.51	-0.10
	FASN	2.61	-0.29	0.64	-0.09
	MCAT	-0.09	-0.15	-0.07	-0.09
	OLAH	1.85	0.98	0.00	0.00
Glycolysis/Gluconeogenesis	ACSS2	0.14	0.15	0.19	0.02
	ADH1	0.81	2.80	1.96	3.14
	ADPGK	0.52	0.33	0.50	0.70
	AKR1A4	-0.18	0.01	0.01	0.00
	ALDH3A1	0.31	-1.01	-1.45	2.70
	ALDH7A1	0.03	-0.92	0.82	-0.75
	ALDOA	-0.12	-0.26	-0.06	-0.16
	BPGM	1.59	-1.24	1.36	0.00
	DLAT	-0.20	-0.10	-0.25	-0.66
	ENO1	-0.40	-0.32	-0.19	-0.25
	FBP2	-1.66	-0.38	2.96	1.55
	G6PC	0.84	1.06	0.65	0.26
	GALM	-0.81	-0.67	0.09	-1.14
	GCK	1.42	-0.95	0.24	-0.60
	GPI1	-0.09	-0.03	0.10	-0.21
	HK1	-0.06	-1.93	-0.53	-0.58
	LDHA	-0.07	-0.21	-0.01	0.03
	PCK1	1.97	0.27	-0.40	0.68
	PDHA1	-0.23	0.05	0.00	0.17
	PFKL	0.20	0.09	0.32	0.17
	PGK1	-0.13	-0.15	-0.36	-0.18
	PGM2	-0.16	-0.10	0.05	-0.09
	PKM2	0.01	0.14	0.28	0.07
	TPI1	-0.05	-0.17	0.00	0.30
Pentose phosphate pathway	DERA	-0.45	-0.60	0.10	-0.49
	GPD2	0.26	0.03	-0.68	-0.38
	H6PD	0.19	0.28	0.26	0.26
	PGD	0.15	0.03	-0.16	-0.07
	PGLS	0.44	0.12	-0.04	0.56
	PRPS2	-0.02	-0.05	-0.37	-0.04
	RBKS	-0.29	0.21	0.15	-0.16
	RGN	-0.36	1.58	2.05	0.11
	RPE	-1.77	-2.27	-0.23	-1.41
	RPIA	-0.17	-0.26	-0.11	-0.17
	TALDO1	-0.16	-0.05	-0.18	-0.22
	TKT	0.28	0.37	-0.25	0.27
	GAPDH	-0.46	-0.33	-0.23	-0.37

Average (log 2)-fold changes in gene expression in sorted $CD4^+PD1^-$, $CD4^+PD1^+$, $CD8^+PD1^-$ and $CD8^+PD1^+$ T cells from spleens of eRapa versus control diet fed mice (3 mice/group).

Table S2. eRapa induces changes in chemokine and chemokine receptor genes in T cells.

	Gene	CD4+PD1-	CD4+PD1+	CD8+PD1-	CD8+PD1+
Chemokines	CCL1	0.00	0.57	0.00	0.00
	CCL2	-0.09	-0.20	-0.25	0.42
	CCL3	-3.17	-3.08	-1.23	-2.43
	CCL4	-4.38	-2.61	-1.17	-1.68
	CCL5	-1.72	-1.81	0.02	-0.73
	CCL6	-1.72	-0.67	0.87	-2.22
	CCL7	0.43	1.05	0.10	-1.21
	CCL9	-2.38	-1.91	-2.52	-3.46
	CCL11	-0.42	2.31	0.75	0.47
	CCL17	-0.75	0.98	0.20	0.65
	CCL19	0.24	0.03	-0.60	0.31
	CCL20	1.25	-1.71	1.25	-0.73
	CCL21A	1.45	-0.07	-0.31	-0.05
	CCL21C	0.00	-0.38	0.00	0.00
	CCL22	0.94	-2.08	-0.99	-0.23
	CCL24	0.00	-0.37	1.18	0.00
	CCL25	0.26	0.06	0.01	1.13
	CCL26	2.16	-0.71	-0.16	0.52
	CCL27	0.74	0.27	0.13	-0.35
	CCL28	0.48	-0.09	-0.16	-2.34
	CXCL1	0.00	-0.43	0.00	0.33
	CXCL2	-0.58	0.01	0.00	-1.62
	CXCL4	-0.90	-0.94	-0.19	-0.50
	CXCL9	-3.00	-1.59	-0.93	-1.03
	CXCL10	-5.74	-0.42	-1.46	-0.26
	CXCL11	0.59	-1.85	3.94	0.21
	CXCL12	-1.25	0.66	0.40	0.75
	CXCL13	1.35	0.06	-2.01	-1.02
	CXCL14	-1.88	0.00	0.00	0.00
	CXCL16	-0.16	-1.81	-1.15	-1.75
	CXCL17	0.00	-0.34	0.49	0.60
	CX3CL1	1.51	0.56	1.29	1.27
	XCL1	-3.33	-1.55	-0.31	-0.28
Chemokine receptors	CCR1	2.05	0.38	-0.92	0.44
	CCR1L1	-1.93	0.36	1.56	-0.22
	IL8RA	1.89	0.58	-0.12	0.56
	IL8RB	0.42	-0.68	-0.56	0.70
	CCR2	0.16	0.00	-0.44	0.00
	CCRL2	-2.08	-0.25	-1.60	-0.78
	CCR3	0.00	0.17	0.00	-0.14
	CCR4	0.20	1.89	0.00	0.00
	CCR5	-5.49	-3.21	-0.75	-1.98
	CCR6	-1.10	1.47	-2.69	-0.76
	CCR7	0.97	1.40	1.05	1.90
	CCR8	-1.79	0.78	0.00	-1.63
	CCR9	1.56	-1.58	0.60	-1.35
	CCR10	1.28	0.66	0.54	-1.09
	CXCR3	-2.15	-0.66	-0.17	-0.60
	CXCR4	0.90	-0.16	0.72	0.17
	CXCR5	-1.79	-1.20	-0.70	-1.16
	CXCR6	-1.26	-0.30	-0.97	-0.54
	CXCR7	1.26	1.54	-0.77	0.00
	CX3CR1	-0.87	-0.92	-0.90	-1.58
	XCR1	0.00	0.00	0.00	0.00

Average (log 2)-fold changes in gene expression in sorted $CD4^+PD1^-$, $CD4^+PD1^+$, $CD8^+PD1^-$ and $CD8^+PD1^+$ T cells from spleens of eRapa versus control diet fed mice (3 mice/group).

Table S3. eRapa induces changes in gene expression in B cells and myeloid cells genes regulating activation and differentiation.

Gene	B220+	CD11b+ CD11c-	CD11b+ CD11c+
ARG1	-0.13	-1.91	0.72
BTLA	1.03	-0.18	0.39
CCL22	0.00	0.00	0.25
CCL3	-2.79	-0.67	-1.07
CCL5	-3.19	-0.73	-1.02
CCR2	2.03	0.00	0.00
CD14	-1.44	1.17	0.79
CD36	0.59	-0.70	-1.20
CD40	1.18	0.03	0.52
CD40LG	0.00	0.84	0.00
CD80	0.55	0.10	0.64
CD86	-0.21	0.42	0.76
CD163	-2.11	1.73	1.54
CD244	-0.91	0.13	-1.64
CD274	0.29	-0.08	0.46
CEBPB	-1.83	0.47	-0.20
CHI3L3 (YM1)	-3.90	0.29	-0.69
CSF1R (CD115)	-2.32	0.72	0.44
CX3CR1	-4.10	0.53	-0.45
CXCL1 (KC)	-2.30	-1.57	-0.51
DPP4 (CD26)	-0.13	-0.02	0.22
EBI3 (IL-35)	0.17	0.40	0.70
EMR1 (F4/80)	-2.99	0.35	0.25
EOMES	-2.68	-1.75	-1.51
FAS	-1.85	-0.33	-0.50
FASL	-2.87	-0.97	-1.21
FCGR1	-1.15	0.09	-0.20
FCGR2B	0.39	-0.30	-0.13
FCGR3	-4.86	0.51	0.14
FLT3	-1.30	-1.11	1.20
GALNTL2	1.72	1.32	-1.04
GATA1	0.00	-1.71	1.33
GATA2	-0.93	-0.44	-0.45
GATA3	-2.87	-1.61	-1.77
H2-AB1	0.41	0.14	0.11
H2-D1	0.36	0.15	0.36
H2-EB1	0.11	-0.10	-0.10
ICOS	-2.52	-0.59	-0.82
ICOSL	1.45	0.33	0.77
IFNA1	0.88	-0.09	0.44
IFNG	-3.37	-1.37	-1.80
IL1A	0.22	-1.38	0.57
IL1B	-2.95	0.82	0.77
IL4	-2.49	-0.32	-1.09
IL6	-0.31	-0.36	-0.12
IL10	-5.02	-1.25	-1.29
IL12A	1.76	-0.37	0.27
IL12B	3.37	1.68	-1.23
IL18	-1.08	0.46	0.44
IL21	-1.01	-3.39	-2.26
IL23A	0.88	-2.48	-0.56
IL33	0.48	-1.11	0.65

Gene	B220+	CD11b+ CD11c-	CD11b+ CD11c+
IL4RA (CD124)	0.46	0.64	-2.54
IL8RA (CXCR1)	-3.47	1.96	2.28
IL8RB (CXCR2)	-2.54	0.88	0.54
INDO (IDO)	-2.18	0.53	0.25
IRF4	0.61	0.00	0.15
ITGA4 (CD49d)	0.53	0.44	0.42
ITGAM (CD11b)	-0.91	0.12	0.46
ITGAX (CD11c)	0.03	-0.26	-1.52
JMJD3	-0.42	0.68	0.36
KIT (CD117)	-3.01	0.30	0.11
KLRE1	-2.70	-0.73	-1.01
KLRG1	-3.31	-1.12	-1.02
LAG3	-6.01	-2.10	-1.74
LY6A	0.07	-0.60	-0.53
LY6C1	-3.60	-0.40	-1.12
LY6D	1.33	-0.08	0.38
LY75 (CD205)	1.27	-1.67	-2.55
MAFB	0.14	0.90	0.68
MERTK	-0.94	1.59	1.12
MIF	-0.03	-0.10	-0.13
MKI67	-2.65	-0.66	-1.42
MMP7	0.00	0.00	0.00
MMP9	-2.36	0.47	-0.02
MPO	-1.60	0.48	-0.36
MRC1 (CD206)	-3.51	0.92	1.07
MTDNA_COXII	-0.76	1.51	-0.35
NCAM1	0.41	0.47	-0.07
NOS1 (nNOS)	1.27	1.53	0.03
NOS1AP	-1.92	1.45	1.60
NOS2 (iNOS)	-0.26	-1.21	-1.24
NOS3 (eNOS)	-1.07	-1.42	-0.80
NOX4	1.58	-2.66	1.20
PTPRC (CD45)	0.06	-0.77	-0.72
RBPJ	-0.43	0.25	-1.63
RETNLB (FIZZ1)	-0.64	0.77	-0.25
SELL (CD62L)	-0.10	-0.30	-0.20
SFPI1 (PU.1)	-0.12	0.45	0.34
SIGLECH	0.30	-0.27	-0.55
STAT1	-0.49	-0.50	-0.58
STAT3	-0.41	0.23	-0.23
STAT6	-0.06	0.33	0.12
TBX21	-2.02	-0.64	-0.94
TEK (TIE2)	0.76	-3.56	2.47
TGFA	0.61	-0.67	-0.16
TGFB1	0.44	0.55	0.35
TIE1	0.60	0.37	-0.59
TNF	-0.41	-0.61	-0.48
VCAM1	-2.93	1.01	0.83
VEGFA	-1.30	-0.79	0.41
VEGFB	0.28	0.02	0.04
VEGFC	-1.60	0.55	-2.43
ZBTB46	1.30	0.51	0.29

Average (log 2)-fold changes in gene expression in sorted $B220^{+}$,$CD11b^{+}CD11c^{-}$ and $CD11c^{+}$ cells from spleens of eRapa versus control diet fed mice (3 mice/group).

Table S4. eRapa induces changes in B cell gene expression.

Gene	$B220^+$
AICDA	-1.31
BACE2	0.00
BCL6	0.29
C1R	-0.60
CD19	1.44
CD27	-2.38
CD38	0.62
CD79B	1.07
CD93 (AA4.1)	-0.91
CR2 (CD21)	3.20
DLL1	-0.61
EBF1	0.86
EBF2	-1.29
EBF3	-0.87
EBF4	0.00
FCER1A	-2.27
FCER1G	-2.43
FCER2A (CD23)	2.29
FGR	-0.77
HCK	0.21
IL2RA	-1.31
IL2RB	-0.16
IL7R	-2.68
ITGAM (CD11b)	-0.91
ITGAX (CD11c)	0.03
KIT	-3.01
LCK	-1.48
LCN10	-0.76
LYN	-0.87
MS4A1 (CD20)	1.49
PAX5	0.15
PECAM1	1.36
PRDM1 (BLIMP-1)	2.02
PRICKLE1	-2.44
RAG1	0.80
RAG2	0.27
RUNX2 (CBF1)	-0.03
SFPI1 (PU.1)	-0.12
SOX5	0.27
SPN (CD43)	-3.20
TCF3 (E2A)	0.48
TNFSF11 (RANKL)	-0.01

Gene	$B220^+$
IGH-4	0.03
IGH-6	-0.10
IGH-V10	0.63
IGH-V11	-0.95
IGH-V15	0.73
IGHA_J00475$V00785_IG_HEAVY_CONSTANT_ALPHA_135	1.61
IGHG	1.19
IGHV1S31_X02463_IG_HEAVY_VARIABLE_1S31_40	1.50
IGHG1_J00453$V00793_IG_HEAVY_CONSTANT_GAMMA_1_792	-1.13
IGHV10S1_AF064442_IG_HEAVY_VARIABLE_10S1_100	3.51
IGHV1S120_AF025443_IG_HEAVY_VARIABLE_1S120_8	-0.94
IGHV1S121_AF025445_IG_HEAVY_VARIABLE_1S121_173	1.29
IGHV1S122_AF025446_IG_HEAVY_VARIABLE_1S122_187	-1.19
IGHV1S123_AF025448_IG_HEAVY_VARIABLE_1S123_197	0.42
IGHV1S124_AF025449_IG_HEAVY_VARIABLE_1S124_11	1.05
IGHV1S129_AF304548_IG_HEAVY_VARIABLE_1S129_110	2.32
IGHV1S133_AF304553_IG_HEAVY_VARIABLE_1S133_89	2.05
IGHV1S135_AF304556_IG_HEAVY_VARIABLE_1S135_43	-1.25
IGHV1S136_AF304557_IG_HEAVY_VARIABLE_1S136_154	-1.30
IGHV1S28_X02460_IG_HEAVY_VARIABLE_1S28_13	-1.66
IGHV1S41_X06868_IG_HEAVY_VARIABLE_1S41_72	-2.15
IGHV8S6_U23021_IG_HEAVY_VARIABLE_8S6_61	-2.78
IGK-C	0.16
IGK-V1	0.17
IGK-V33	0.82
IGK-V38	0.55
IGK-V5	1.08
IGKV1-88_AJ231206_IG_KAPPA_VARIABLE_1-88_289	1.17
IGKV1-99_AJ231207_IG_KAPPA_VARIABLE_1-99_1	-2.21
IGKV4-75_AJ231227_IG_KAPPA_VARIABLE_4-75_15	1.16
IGKV4-80_AJ231213_IG_KAPPA_VARIABLE_4-80_91	-0.16
IGKV4-90_AJ231224_IG_KAPPA_VARIABLE_4-90_22	1.07
IGKV4-91_AJ231229_IG_KAPPA_VARIABLE_4-91_29	0.76
IGKV6-13_J00569_IG_KAPPA_VARIABLE_6-13_23	-1.52
IGKV8-31_AJ235957_IG_KAPPA_VARIABLE_8-31_3	0.46
IGKV9-120_V00804$J00566_IG_KAPPA_VARIABLE_9-120_12	0.67
IGKV9-128_AJ231245_IG_KAPPA_VARIABLE_9-128_15	0.79
IGKV12-98_AJ235949_IG_KAPPA_VARIABLE_12-98_12	1.36
IGL-5	1.06
IGL-V1	0.85
IGLC2_J00595_IG_LAMBDA_CONSTANT_2_14	0.44
IGLJ4_J00596_IG_LAMBDA_JOINING_4_7	0.09

Average (log 2)-fold changes in gene expression in sorted $B220^+$ B cells from spleens of eRapa versus control diet fed mice (3 mice/group).

Table S5. eRapa induces changes in apoptosis and inflammasome related gene expression.

	Gene	CD4+PD1+	CD4+PD1-	CD8+PD1+	CD8+PD1-	B220+	CD11b+CD11c-	CD11c+
Apoptosis	AIP	0.05	0.16	-0.01	0.11	0.01	0.12	-0.01
	BAK1	-2.61	-1.96	1.60	-1.52	-0.62	-0.52	-1.45
	BAX	0.06	0.44	-0.37	-0.16	0.19	0.01	0.22
	BCL2	0.49	0.58	0.12	0.18	0.08	-0.32	-0.10
	BCL2L1	1.40	0.60	-2.16	-0.16	-0.27	-0.67	-2.00
	BID	0.05	0.15	-0.13	-0.29	0.17	0.38	0.25
	BIRC2	-0.08	-0.30	0.00	0.03	0.29	-0.14	0.17
	BIRC3	0.00	0.00	0.00	0.00	-0.56	-1.01	0.28
	BIRC5	-0.49	-1.37	-0.99	-1.35	-1.21	-0.42	-0.76
	BIRC6	-0.34	0.52	1.09	-0.85	0.88	-0.60	0.40
	BIRC7	-0.54	0.03	-0.04	0.64	1.27	0.36	0.98
	CASP3	-1.61	-3.68	-3.65	-1.47	-0.35	-0.33	-2.57
	CASP7	-0.79	-1.32	-0.38	-0.33	-0.08	0.00	-0.01
	CASP8	0.35	-0.14	-0.26	-0.08	0.02	-0.24	-0.30
	CASP9	-0.17	0.21	-0.29	0.03	0.31	0.03	0.14
	DFFA	0.64	-0.58	0.27	0.04	0.15	0.00	-0.09
	DFFB	-1.31	1.05	0.68	0.64	-1.71	0.07	1.06
	DIABLO	0.34	0.23	0.05	0.07	0.41	0.04	0.36
	FADD	-0.16	-0.02	0.27	-0.01	-0.07	-0.10	-0.05
	FAS	-0.07	-0.37	-0.10	0.51	-1.85	-0.33	-0.50
	FASL	-1.06	-1.09	-1.34	-0.55	-2.87	-0.97	-1.21
	HTRA2	-0.17	-0.30	-0.25	-0.02	0.32	0.05	0.03
	IAP	-0.21	0.05	0.54	0.26	-0.50	-0.05	0.01
	MCL1	0.05	0.13	-0.15	-0.18	-0.08	0.11	-0.06
	NAIP1	0.42	0.79	0.12	-0.12	0.53	0.24	-0.67
	NAIP2	-1.81	-1.53	-2.93	-0.66	-1.39	0.21	-1.00
	RIPK1	-0.29	-0.24	-0.41	0.02	-0.52	-0.11	-0.02
	TNFRSF10B (DR5)	0.46	-0.75	-1.17	0.77	-0.09	1.12	-0.85
	TNFRSF1A (TNF-R1)	-0.66	-0.68	-0.84	-0.29	-3.25	0.52	-0.77
	TNFRSF6 (FADD)	-0.96	-2.12	-1.07	2.37	0.00	0.31	-1.02
	TRADD	-1.25	-1.10	-0.69	-0.43	0.97	-0.56	-0.35
	TRP53	0.23	0.29	0.41	0.31	0.93	0.19	0.07
Inflammasome	AIM2	-0.41	-0.38	-0.17	-0.70	0.57	-0.11	-0.17
	CASP1	-0.35	-1.23	-0.77	-0.76	0.12	-0.15	-0.16
	CASP4	-0.19	-0.74	-0.58	-0.60	-0.38	-0.13	-0.52
	IL18	-1.49	-3.04	-2.18	-2.80	-1.08	0.46	0.44
	IL1A	-1.18	1.76	-1.23	-2.56	0.22	-1.38	0.57
	IL1B	-1.93	-2.51	-3.95	-0.62	-2.95	0.82	0.77
	NAIP5	-0.01	-4.51	-1.32	-0.83	-2.77	0.18	-0.16
	NLRC4	-0.80	0.71	0.16	0.22	1.01	0.49	0.86
	NLRP1C	-1.01	0.00	-1.05	-0.05	-0.77	0.00	0.12
	NLRP3	0.88	0.81	0.46	0.73	-0.93	-1.43	0.81
	PYCARD	-0.92	-0.18	-0.30	-1.02	-1.60	-0.30	-1.20

Average (log 2)-fold changes in gene expression in sorted cells from spleens of eRapa versus control diet fed mice (3 mice/group).

Table S6. eRapa induces changes in mTOR regulated and autophagy gene expression.

	Gene	CD4+PD1+	CD4+PD1-	CD8+PD1+	CD8+PD1-	B220+	CD11b+CD11c-	CD11c+
mTOR	ACLY	-0.29	-0.06	0.09	-0.07	-0.31	-0.15	-0.29
	ATP6V0D2	-0.53	-0.41	-0.15	-1.43	-1.40	-0.25	-1.61
	BIRC5	-0.49	-1.37	-0.99	-1.35	-1.21	-0.42	-0.76
	CCND1	0.02	-0.17	0.02	-0.23	-1.24	0.51	0.43
	CEBPA	0.08	0.88	0.73	0.00	-0.83	-0.79	-0.04
	CTSD	-1.50	-0.75	-2.25	0.14	-0.52	0.25	0.66
	EEF1A1	-0.03	-0.07	-0.02	0.01	0.20	-0.13	0.05
	EEF2K	0.77	0.79	3.43	-0.50	-1.40	0.08	0.27
	EIF4B	-1.29	-0.33	-0.45	-0.18	0.02	-0.92	-1.40
	EIF4E	0.46	-0.12	-0.15	0.45	0.57	-0.43	0.58
	FABP4	1.62	0.46	0.49	-0.75	-0.79	0.00	-0.42
	FASN	-0.29	2.61	-0.09	0.64	3.59	1.17	1.22
	HIF1A	-2.36	-3.51	-0.15	-1.46	-0.98	-3.34	-0.49
	HK1	-1.93	-0.06	-0.58	-0.53	1.03	-0.65	-0.74
	LAMP1	-0.31	0.03	0.09	0.06	-0.53	0.31	0.22
	MT-ATP6	-0.27	0.25	0.06	-0.23	-2.64	0.21	0.15
	MYC	-0.62	-2.23	-0.71	-0.54	0.43	-0.78	1.21
	RBPJ	2.19	2.34	2.68	-0.06	-0.43	0.25	-1.63
	RPS6	-0.02	-0.06	0.24	0.10	0.37	0.00	-0.10
	RPS6KB1	-0.13	-1.15	0.79	0.12	0.61	-0.55	1.19
	RPS6KB2	-3.64	-1.95	-0.81	-0.45	1.35	-0.53	-2.20
	S6K	-0.28	-1.94	2.61	1.47	1.19	0.73	-2.10
	SREBF2	-0.13	0.21	0.25	0.22	0.71	-0.03	0.05
	TWIST1	-0.38	1.08	1.04	0.51	-0.63	-0.55	0.36
	TWIST2	0.88	0.00	0.00	0.00	0.00	0.00	-1.59
	VEGFA	0.00	0.00	0.00	0.00	-1.30	-0.79	0.41
	VIM	0.31	-0.79	-0.37	-0.45	-0.70	-0.65	-0.65
Autophagy	ATG10	0.11	-0.13	0.27	0.20	-0.08	-0.14	0.13
	ATG12	-0.21	-0.31	-0.15	-0.04	-0.16	0.02	0.10
	ATG16L1	0.15	-0.06	0.22	0.25	0.40	0.35	0.35
	ATG16L2	-0.09	-0.05	-0.13	0.13	0.88	0.14	0.17
	ATG2A	0.20	0.00	0.11	0.30	-0.01	0.48	0.22
	ATG2B	0.03	0.19	0.37	0.17	0.11	0.06	0.15
	ATG3	-0.12	-0.31	-0.06	-0.25	-0.64	-0.06	-0.44
	ATG4B	0.17	-0.98	0.44	-0.72	-0.41	0.13	0.12
	ATG4C	2.18	2.61	-1.17	-0.92	1.47	0.67	1.12
	ATG4D	-0.09	0.10	0.44	-0.06	0.89	0.23	0.25
	ATG5	0.10	0.37	0.00	0.04	-0.32	-0.04	0.00
	ATG7	0.09	0.85	0.66	-0.22	-0.68	0.50	0.23
	ATG9A	-0.49	1.00	0.29	0.02	-1.29	0.16	-0.07
	ATG9B	-1.54	-2.31	1.57	3.66	-3.56	-0.22	-1.10
	BAG1	0.06	-0.11	-0.23	0.00	-0.29	-0.06	0.05
	BAG2	0.39	0.92	0.47	-1.19	0.99	-0.28	1.15
	BAG3	-0.62	-1.21	-0.69	-0.07	-2.15	-0.03	-0.34
	BAG4	-0.54	0.08	-0.29	-0.10	-0.40	-0.02	-0.19
	BECN1 (ATG6)	-0.45	-0.44	-0.06	0.21	0.90	-0.35	-0.06
	GABARAP	-0.21	-0.32	-0.29	-0.08	-0.15	-0.09	-0.15
	HSPA8	-0.13	-0.26	-0.15	-0.05	-0.08	-0.16	-0.06
	INS1	-0.13	1.63	-0.55	-0.20	0.01	0.36	-1.29
	MAP1LC3A (ATG8E, LC3A)	-0.28	-1.12	-0.59	0.86	-1.20	-0.52	-0.47
	MAP1LC3B (ATG8B, LC3B)	-1.02	0.05	-0.86	0.02	-1.81	-0.06	-0.37
	PIK3C3 (VPS34)	-0.18	-0.07	-0.31	0.02	-0.04	-0.17	-0.03
	PIK3R4	-1.04	1.55	-0.69	-0.02	1.52	1.41	0.68
	PRKAA1 (AMPK)	0.65	-1.74	0.55	0.37	-0.27	-0.48	0.12
	ULK1 (ATG1)	-0.52	-0.20	0.02	0.39	-0.02	0.68	-0.25

Average (log 2)-fold changes in gene expression in sorted cells from spleens of eRapa versus control diet fed mice (3 mice/group).
